# Mechanisms underlying the mitigating action of Maxing Shigan Decoction in acute lung injury caused by influenza virus based on UPLC-HRMS analysis and network pharmacology

**DOI:** 10.3389/fimmu.2025.1634442

**Published:** 2025-09-01

**Authors:** Jue Hu, Xiangming Ma, Yufeng Xiao, Chunjing Chen, Chang Liu, Jun Lu, Xiaoqi Wang, Fangguo Lu

**Affiliations:** ^1^ Medical School, Hunan University of Chinese Medicine, Changsha, China; ^2^ School of Integrated Chinese and Western Medicine, Hunan University of Chinese Medicine, Changsha, China

**Keywords:** Maxing Shigan Decoction, acute lung injury, UPLC-HRMS, blood-absorbed components, macrophage polarization

## Abstract

**Introduction:**

Influenza A virus (IAV) infection is associated with high morbidity and mortality and can ultimately lead to acute lung injury (ALI). In traditional Chinese medicine, Maxing Shigan Decoction (MXSGD) can treat exogenous wind-cold, toxic heat invading the lungs, and heat-toxicity obstructing the lungs. However, the active components and underlying mechanisms of MXSGD in IAV-induced diseases remain largely unexplored. Therefore, we aimed to investigate the active constituents of MXSGD and its underlying mechanism of action in ALI.

**Methods:**

Bioactive components of MXSGD in rat serum were identified using ultra-high-performance liquid chromatography and high-resolution mass spectrometry (UPLC-HRMS). Blood-absorbed MXSGD components (i.e., the constituents of MXSGD detectable in serum) in ALI were predicted through network pharmacology and molecular docking analyses. A mouse lung injury model was established using the influenza virus. The degree of lung injury, viral load in lung tissues, serum levels of inflammatory factors, gene expression levels of inflammation-related factors in lung tissue, and macrophage polarization in the lungs were then assessed.

**Results and discussion:**

In the rat serum, 242 bioactive components were identified using UPLC-HRMS. Moreover, 56 ingredients, including glycyrrhizin, amygdalin, and ephedrine, were analyzed using network pharmacology, revealing 338 ALI-related targets and 99 core proteins in the protein–protein interaction network. Gene Ontology and Kyoto Encyclopedia of Genes and Genomes pathway analyses were conducted for core targets, and molecular docking confirmed the binding affinity of the main identified targets with their respective blood-absorbed components. Validation results demonstrated that MXSGD significantly ameliorated lung injury, mitigated lung congestion and inflammation, lowered viral load in mouse lung tissue, promoted macrophage polarization, and downregulated the expression of the PI3K/AKT pathway in IAV-infected mice. Overall, this study revealed the mechanisms and active ingredients underlying the therapeutic effects, highlighting of MXSGD its potential in treating IAV-induced ALI and regulating the polarization of macrophages.

## 1 Introduction

Influenza is a major cause of respiratory infections worldwide and is associated with high morbidity and mortality rates, often leading to complications such as pneumonia. Without timely treatment, these infections can progress to acute lung injury (ALI) and acute respiratory distress syndrome (ARDS), a leading cause of death in severe cases. According to the World Health Organization, seasonal influenza results in 3–5 million cases of severe illness and between 290,000 and 650,000 deaths annually (1).

Traditional Chinese medicine (TCM) has demonstrated notable efficacy in the treatment of infectious diseases (2, 3). Certain TCMs and their active components can effectively prevent and treat acute lung damage, and the preventive benefits of TCM in mitigating lung injury are garnering increasing recognition. For example, puerarin can significantly mitigate ALI by activating liver X receptor alpha and attenuating lipopolysaccharide (LPS)-induced inflammatory responses (4). Moreover, treatment with the Huayu Lifei formula showed decreased expression of tumor growth factor-beta (TGF-β), connective tissue growth factor, and Smad3 proteins in rats (5). Maxing Shigan Decoction (MXSGD), derived from *Shang Han Lun*, is a classic TCM treatise compiled by Zhang Zhongjing. It is commonly used to promote lung ventilation, liminate evil heat, clear inflammation, and relieve respiratory conditions such as cough, bronchitis, pneumonia, and asthma. MXSGD is also widely used to treat syndromes characterized by toxic heat invading the lungs and heat-toxicity obstructing lung function (6, 7). During the COVID-19 pandemic, MXSGD and its derivatives were frequently included in Chinese health guidelines as treatments for viral pneumonia (8, 9). Notably, MXSGD exerts inhibitory effects against multiple subtypes of the influenza virus, including H1N1, H9N2, H6N2, and type B influenza. MXSGD is commonly used to treat patients with pathogenic wind invasion at the body surface and heat-toxicity obstructing the lungs. The 2018–2020 Guidelines for the Diagnosis and Treatment of Influenza, issued by the National Health Commission of China, included this formula as a core treatment for the syndrome characterized by heat-toxicity blocking the lungs (10–13). The antiviral action of MXSGD may involve suppressing pathogen proliferation, reducing virus-induced inflammation, preventing inflammatory cytokine storms, and ameliorating gut microbial dysbiosis (14–16). However, the complex composition of the active compounds in MXSGD complicates efforts to fully unravel the precise mechanisms underlying its therapeutic effects, particularly in influenza-induced ALI.

**Figure 1:**
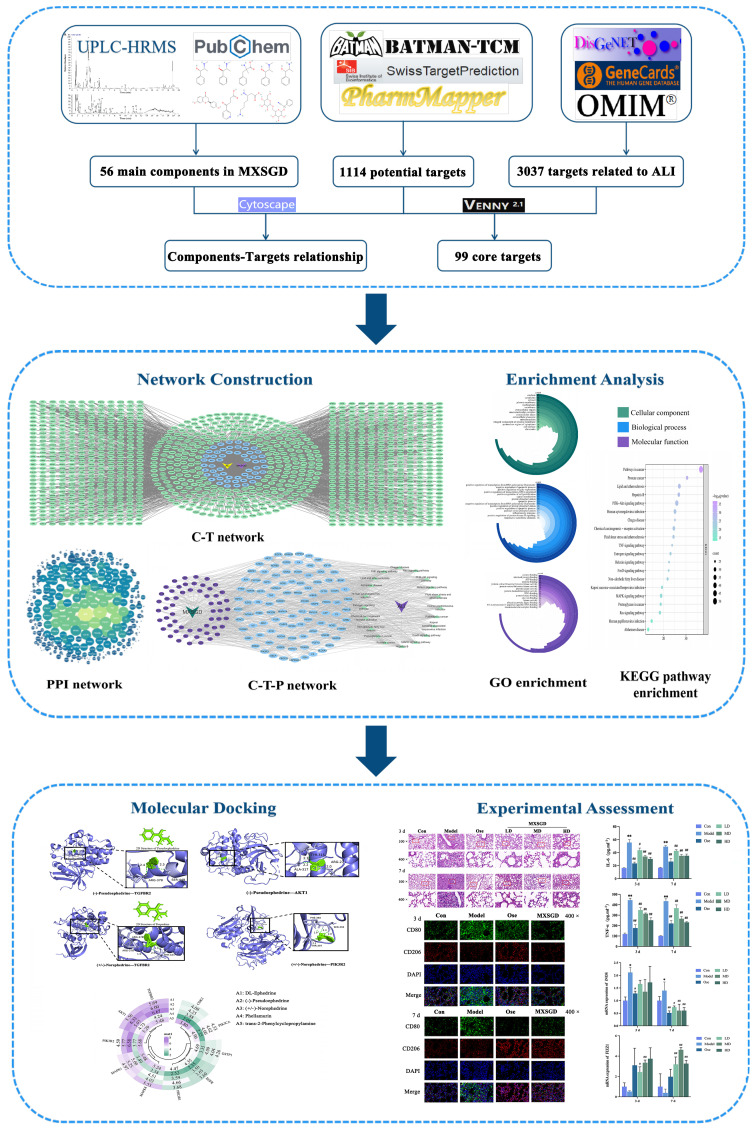
Workflow of systems pharmacology analysis and experimental assessment.

Network pharmacology offers a systematic approach to predict the potential mechanisms underlying the therapeutic effects of herbal formulations by analyzing compound–target–disease interaction networks through bioinformatics. This approach effectively explores the relationships between TCM and diseases, aligning with the holistic perspective of TCM. In the present study, using network pharmacology, we aimed to examine the mechanisms underlying MXSGD activity in ALI, predict the active compounds using ultra-high-performance liquid chromatography-high-resolution mass spectrometry (UPLC-HRMS), and validate the associated pathways through *in vivo* tests (**Figure 1**).

## 2 Materials and methods

### 2.1 Instruments, drugs, and reagents

#### 2.1.1 Instruments

The following instruments were used: a low-temperature high-speed centrifuge (Eppendorf Centrifuge 5430 R; Eppendorf, Hamburg, Germany), Vanquish UHPLC system (Thermo Fisher Scientific, Waltham, MA, USA), ACQUITY UPLC HSS T3 chromatographic column (2.1 mm ×100 mm, 1.8 µm), Q-Exactive HFX mass spectrometer (Thermo Fisher Scientific), ELX-800 multifunctional microplate reader (Bio Tek, Winooski, VT, USA), LightCycler 96 fluorescent quantitative PCR instrument (Roche, Basel, Switzerland), tissue optical scanning microscope camera (ZEISS, Oberkochen, Germany), Mini-PROTEAN vertical electrophoresis apparatus, Mini Trans-Blot transfer apparatus, and SCG-W2000 chemiluminescence imaging system (all from Servicebio, Hubei, China).

#### 2.1.2 Drugs and reagents

The reagents utilized in liquid chromatography-tandem mass spectrometry (LC-MS) included water (MS grade, MilliporeSigma, Burlington, MA, USA), methanol and acetonitrile (MS grade, Thermo Fisher Scientific), and formic acid (MS grade, Honeywell International Inc., Charlotte, NC, USA).

MXSGD consists of four TCM components: Ephedrae Herba (Lot No. 2109170042), Armeniacae Semen Amarum (Lot No. 2022011201), Gypsum fibrosum (Lot No. 2110151), and Glycyrrhizae Radix et Rhizoma (Lot No. 220201). All Chinese medicinal materials were purchased from the First Affiliated Hospital of Hunan University of Traditional Chinese Medicine, and their quality met the standards established by the *Chinese Pharmacopoeia* (2020 edition). Oseltamivir phosphate capsules (75 mg, Lot No. M1073; Roche) were purchased from the People’s Hospital of Hunan Province. The virus strain A/PR/8/34 (H1N1, mouse lung-adapted) was provided by the Molecular Virology Laboratory at Hunan Normal University and amplified in chicken embryos in a BSL-2 laboratory at Hunan University of Chinese Medicine.

The reagents used in the validation experiments included influenza A virus (IAV) nucleoprotein antibody (Cat No. GTX125989; GeneTex, Irvine, CA, USA), anti-CD80 antibody (Cat No. ab254579; Abcam, Cambridge, UK), anti-mannose receptor antibody (Abcam, Cat No. ab64693), HRP peroxidase-conjugated goat anti-rabbit IgG (H+L, Cat No. GB21303; Servicebio), Cy3 conjugated goat anti-rabbit IgG (H+L, Cat No. GB23303; Servicebio), PI3K p85 alpha antibody (Cat No. AF6241; Affinity Biosciences, Cincinnati, OH, USA), phospho-pan-AKT1/2/3 antibody (Cat No. AF3262; Affinity Biosciences), pan-AKT1/2/3 antibody (Cat No. AF6261; Affinity Biosciences), RIPA lysis buffer (Cat No. G2002-100ML; Servicebio), phosphatase inhibitor (Cat No. G2007-1ML; Servicebio), BCA protein quantification assay kit (Cat No. G2026-200T; Servicebio), prestained protein marker VII (8–195 kDa; Cat No. G2087-250UL; Servicebio), enhanced chemiluminescent reagent kit (Cat No. G2161-200ML; Servicebio), RNA simple total RNA extraction kit (Cat No. DP419; TIANGEN, Beijing, China), NovoScript Plus All-in-One 1st Strand cDNA Synthesis SuperMix (gDNA Purge; Cat No. E047-01B; Novoprotein, Shanghai, China), NovoScript SYBR qPCR Super Mixture (Cat No. E096-01A; Novoprotein), mouse IL-1β enzyme-linked immunosorbent assay (ELISA) kit (Cat No. ml301814; MLBIO, Shanghai, China), mouse IL-6 ELISA kit (Cat No. ml063159; MLBIO), and mouse TNF-α ELISA Kit (Cat No. ml002095; MLBIO).

### 2.2 Drug and sample preparation

#### 2.2.1 Preparation of experimental drugs

The MXSGD formulation comprised 9 g of ephedra, 9 g of apricot kernel, 18 g of gypsum, and 6 g of licorice. The medicinal substances were measured according to their compositional ratios during production. The aqueous extract of MXSGD was obtained by first boiling ephedra, following the traditional preparation described in “Shang Han Lun” and based on our previous findings (17). Ephedra was mixed with distilled water at a 1:10 weight-to-volume ratio. The mixture was first boiled at 100°C and then simmered for 25 min. Following the removal of froth, gypsum, apricot kernel, and licorice were added to the mixture, which was then boiled for an additional 30 min and subsequently filtered. For the second extraction, distilled water at seven times the original volume was added, brought to a boil at 100°C, simmered for 20 min, and filtered again. The two extracts were then combined and concentrated to yield final crude drug concentrations of 0.605 g/mL and 4.265 g/mL for animal validation and serum preparation, respectively. The extracts were shielded from light and stored at 4°C until use. Oseltamivir phosphate capsules were completely dissolved in distilled water to create a suspension with a concentration of 2.15 mg/mL.

#### 2.2.2 Preparation of drug-containing serum

Forty-eight specific-pathogen-free (SPF) male Sprague Dawley rats (180–220 g) were purchased from Hunan SJA Laboratory Animal Co., Ltd. [Animal Quality Certificate No. SYXK (Xiang) 2019-0009; Animal Experiment Ethical Approval No.: LL2021081101]. They were housed in the Animal Experiment Center of the Hunan University of Chinese Medicine and allowed to acclimate for two days. The experimental conditions included a 12-h light/dark cycle, unlimited access to water and food, and controlled temperature and humidity. Animals were randomly assigned to two groups: the MXSGD and control groups, each including seven rats. Following serum pharmacochemistry protocols, the MXSGD group received a dosage adjusted based on body surface area to increase the concentration of active drug components in the blood. The gavage dosage was equivalent to 10 times the standard clinical dosage. Each rat in the MXSGD group received a gavage dose of 8.53 g/day. The control group received an equivalent volume of physiological saline, delivered daily at 2 mL for 7 consecutive days. On day 7, after an 8-h fasting period with unlimited access to water, the rats were anesthetized with sodium pentobarbital, and blood was drawn from the abdominal aorta. Blood samples were incubated at 4°C for 2 h and then centrifuged at 3,000 rpm for 10 min. The obtained MXSGD-containing serum and blank serum were stored at –20°C until use.

#### 2.2.3 Preparation of the test solution

A total of 600 μL of MXSGD aqueous extract was mixed with 400 μL of methanol and vortexed. To dilute the mixture, 200 μL of it was added to 1.4 mL of a 40% aqueous methanol solution. The mixture was vortexed once more and centrifuged at 16,000 ×*g* for 15 min at 4°C. The supernatant was collected as the test solution sample (MXSGD-PRE).

#### 2.2.4 Preparation of serum samples

Approximately 200 μL of blank rat serum and 200 μL of MXSGD-containing serum were each combined with 800 μL of methanol. The two solutions were vortexed for 60 s, incubated at -20°C for 30 min, and subsequently centrifuged at 16,000 ×*g* for 20 min at 4°C. The supernatant was collected and vacuum-dried. The residue was dissolved in 100 μL of a 40% aqueous methanol solution, vortexed, and centrifuged again at 16,000 ×*g* for 15 min at 4°C. The supernatant was obtained, yielding a blank serum sample (CONTROL) and a serum sample containing MXSGD (MXSGD).

#### 2.2.5 Preparation of blank serum and test solution samples

Next, 200 μL of blank rat serum was mixed with 33.4 μL of MXSGD aqueous extract. Subsequently, 800 μL of methanol was added to the mixture, which was then vortexed for 60 s. The solution was incubated at –20°C for 30 min, followed by centrifugation at 16,000 ×*g* for 20 min at 4°C. The supernatant was then harvested and vacuum-dried. The residue was subsequently dissolved in 100 μL of a 40% aqueous methanol solution, vortexed, and centrifuged again at 16,000 ×*g* for 15 min at 4°C. The supernatant was obtained, yielding a sample consisting of the blank serum combined with the test solution (CONTROL + MXSGD-PRE).

### 2.3 Application of UPLC-HRMS in pharmaceutical analysis

UPLC-HRMS was used to determine the chemical constituents of MXSGD that reached the bloodstream, with each constituent accurately characterized. Chromatographic separation was performed using a Vanquish UHPLC system equipped with an ACQUITY UPLC HSS T3 chromatographic column (2.1 × 100 mm, 1.8 µm). An aqueous solution of 0.1% formic acid served as mobile phase A, and an acetonitrile solution with 0.1% formic acid functioned as mobile phase B. Gradient elution was conducted as follows: 0–17 min, 5–98% B; 17–17.2 min, 98–5% B; and 17.2–20 min, 5% B. The flow rate was set at 0.3 mL/min, with the column temperature set to 35°C and an injection volume of 2 μL.

The Q-Exactive HFX mass spectrometer was integrated with the UHPLC system, and MS was conducted in both positive and negative modes using electrospray ionization. The data-dependent acquisition mode was utilized to select the top 10 MS1 ions to acquire the MS/MS spectra. Collision energies were established at normalized values of 20, 40, and 60, with a data acquisition range of m/z 90–1300. The spray voltages were set to +3800 V and –3000 V. The sheath gas flow rate was maintained at 45, with the capillary temperature set to 320°C and the probe heater temperature to 370°C. XCMS software was used for peak alignment, retention time correction, and peak extraction. The resulting data obtained were compared with a standard spectrum database for structural identification.

The correlation of the secondary mass spectrum (MS2) was predominantly evaluated using the MS2 fragment score, which has a maximum value of 1 (18, 19). A higher score (i.e., > 0.7) indicates greater reliability of the identification results (20, 21). Consequently, the parameters were set to maintain an MS1 difference of less than 15 ppm and a high degree of MS2 fragment similarity.

### 2.4 Network pharmacology analysis of MXSGD in the treatment of ALI

#### 2.4.1 Screening of the blood-absorbed components and action targets of MXSGD

Representative components of MXSGD that entered the bloodstream, as detected via UPLC-HRMS, were selected as research subjects based on the average ion abundance values. Component IDs were obtained from the PubChem database (https://pubchem.ncbi.nlm.nih.gov/). Probable matching and docking *in vivo* drug targets were predicted using the PharmMapper database (http://www.lilab-ecust.cn/pharmmapper/), with targets filtered based on a Norm Fit threshold of ≥ 0.8. In the SwissTargetPrediction database (http://www.swisstargetprediction.ch), targets were selected based on a “probability” threshold of ≥ 0.2. Targets underwent additional screening using the BATMAN-TCM database (http://bionet.ncpsb.org.cn/batman-tcm/) with a score cutoff” ≥ 20 and a P-value ≥ 0.05. The target protein names were standardized using the UniProt database (https://www.uniprot.org/), and duplicate targets were consolidated and eliminated, yielding the action targets of the components of MXSGD that entered the bloodstream.

#### 2.4.2 Screening of potential targets for ALI

The search term “Acute Lung Injury” was employed to extract pertinent targets from the GeneCards (http://www.genecards.org/), DisGeNET (https://www.disgenet.org/), TTD (https://db.idrblab.net/ttd/), and OMIM databases (http://www.omim.org/). Duplicate targets were consolidated and eliminated, and the target nomenclature was accurately corrected using the UniProt database to identify disease-associated targets. Venny 2.1 (https://bioinfogp.cnb.csic.es/tools/venny/index.html) was subsequently used to input the action targets of the components and diseases, producing a Venn diagram. The intersecting targets indicated the probable action sites of the MXSGD components in the treatment of ALI.

#### 2.4.3 Construction of component–target and protein–protein interaction network diagrams

Based on the identified MXSGD components that entered the bloodstream, target prediction outcomes, and associated illness targets, the C–T network diagram of MXSGD was generated employing Cytoscape 3.7.1 software. The PPI network was constructed using the STRING database (https://cn.string-db.org), with a high confidence threshold (≥ 0.7) as the screening criterion. The resulting network was visualized and analyzed using Cytoscape software (version 3.7.1). The network was further evaluated using the “Network Analyzer” tool, and network topology analysis was performed using CytoNCA (version 2.1.6). Key targets were identified based on degree, betweenness, and closeness centrality metrics, with values exceeding the respective medians.

#### 2.4.4 Gene ontology and Kyoto encyclopedia of genes and genomes pathway enrichment analyses

The potential targets were imported into the Database for Annotation, Visualization and Integrated Discovery (DAVID) database (https://davidbioinformatics.nih.gov/summary.jsp) to conduct GO enrichment analysis for biological function and KEGG pathway enrichment analysis. These analyses highlighted the molecular functions, biological processes, cellular components, and metabolic pathways of these genes. A significance criterion of *P* < 0.05 was set, and part of the results were visualized using the Internet Bioinformatics tool (https://www.bioinformatics.com.cn/).

#### 2.4.5 Construction of the blood-absorbed components–key targets–signaling pathways network

The blood-absorbed components of MXSGD, target prediction outcomes, pathway analysis, and associated diseases were utilized to delineate the relationships among drugs–ingredients, ingredients–targets, targets–pathways, and pathways–diseases. A network diagram titled “Blood-Absorbed Components –Key Targets–Signaling Pathways” (CTP) was generated utilizing Cytoscape software (version 3.7.1).

#### 2.4.6 Molecular docking

The top five potentially active components with a high degree from the CTP network were selected for molecular docking with the top 10 core targets. The structures of prospective active constituents were acquired from the PubChem database. PDB format files for the principal targets were retrieved from the PDB database (https://www.rcsb.org). Hydrogenation, dehydration, and ligand separation were performed using the AutoDock Tools software (version 1.5.7). Molecular docking was performed using AutoDock Vina software (version 1.1.2), and the minimal binding energies were computed. The docking findings were imported into PyMol for visualization and graphing.

### 2.5 Animal experiments

#### 2.5.1 Establishment of animal models, grouping, and medication

Overall, 48 SPF-grade BALB/c mice (16–20 g), equally divided between males and females, were purchased from Hunan SJA Laboratory Animal Co., Ltd. [Animal Quality Certificate No. SYXK (Xiang) 2020-0010; Animal Experiment Ethical Approval No.: ZYFY20230703-04). The rearing conditions comprised a 12-h light/dark cycle, unlimited access to water and food, and controlled temperature and humidity levels. All animals were randomly allocated to six groups: control, model, positive control medicine (oseltamivir, Ose), and low-dose, medium-dose, and high-dose MXSGD groups, with eight mice in each group. All groups, excluding the control group, received 0.05 mL of a 1:640 hemagglutination titer dilution of IAV (1:100) by nasal drops to establish the IAV infection model. Twenty-four hours post-infection, each group received the corresponding treatment via oral gavage at clinically equivalent doses, using a body surface area-based dose variation algorithm. The medication was administered once daily at a volume of 0.2 mL. In the positive control treatment group, each mouse received 0.433 mg of oseltamivir daily by gavage. The low, medium, and high MXSGD groups received daily doses of 0.0605, 0.121, and 0.242 g of MXSGD, respectively. An equivalent volume of physiological saline was administered to the control and model groups. After 3 or 7 consecutive days of treatment, the animals were weighed, and blood was collected from the eyeball after anesthesia. Lung tissues of the mice were excised and weighed, with a portion preserved in 4% paraformaldehyde and another portion maintained in a –80°C ultra-low-temperature freezer.

#### 2.5.2 General observation of mice

At the end of the experiment, the body weights of the mice were recorded. The lungs were harvested, and the remaining blood was absorbed using filter paper prior to weighing and documenting the lungs to compute the organ index. The lung index (%) was calculated using the following formula:


$$Lung\;Index\;\left(\%\right)\;=\;\left(Lung\;Weight\;\left(g\right)/Body\;Weight\;\left(g\right)\right)\;\times \;100$$

#### 5.3 Pathological observation of lung tissue

Lung tissues preserved in 4% paraformaldehyde were subjected to gradient alcohol dehydration, xylene clearing, paraffin embedding, sectioning, baking, deparaffinization, hematoxylin-eosin staining, and neutral resin mounting. Tissue samples were examined under a microscope, and pathological changes were documented using photography.

#### 2.5.4 Immunofluorescence detection of IAV nuclear protein levels in lung tissue

Three mice per group were randomly selected for immunofluorescence detection. After embedding, lung tissue samples were sectioned and deparaffinized for antigen retrieval. The tissue sections were blocked with 3% bovine serum albumin (BSA) for 30 min and then incubated with IAV nucleoprotein antibody (1:400) overnight at 4°C. Subsequently, they were incubated with a CY3-conjugated secondary antibody (1:300, red) at room temperature for 50 min. Nuclear staining was then performed using 4′,6-diamidino-2-phenylindole (DAPI) (blue) in the dark at room temperature for 10 min. Following the suppression of tissue autofluorescence using an autofluorescence quencher, an anti-fluorescence quenching mounting solution was used, and the samples were examined under a fluorescence microscope. Image-Pro Plus 6.0 was then used to analyze the acquired images and determine the mean optical density value (IOD/area) for each field of view.

#### 2.5.5 ELISA detection of cytokine levels in mouse serum

Frozen mouse serum was retrieved, and the levels of cytokines (IL-1β, IL-6, and TNF-α) were measured according to the instructions of the ELISA kit. After obtaining absorbance values, sample concentrations were calculated using a standard curve.

#### 2.5.6 Reverse transcription quantitative polymerase chain reaction of the gene expression of macrophage polarization markers in pulmonary tissue

Total RNA was extracted from pulmonary tissue using the TIANGEN RNAsimple Total RNA Kit. The extracted total RNA was then reverse-transcribed into cDNA using the NovoScript^®^ Plus All-in-one 1st Strand cDNA Synthesis SuperMix (gDNA Purge). Subsequently, PCR amplification was performed using the NovoStart^®^ SYBR qPCR SuperMix Plus kit. Following the PCR reaction, melting curve analysis was conducted, and the relative expression levels of target genes were calculated using the 2−^ΔΔCT^ method, followed by statistical analysis. The specific primers utilized are presented in **Table 1**.

**Table 1:** Primer sequences.

Genes	Primer sequences(5’—3’)
*IL-6*	F: GACTTCCATCCAGTTGCCTT
	R: ATGTGTAATTAAGCCTCCGACT
*IL-10*	F: GGACAACATACTGCTAACCGACTC
	R: TGGATCATTTCCGATAAGGCTTGG
*iNOS*	F: TCACTCAGCCAAGCCCTCAC
	R: TCCAATCTCTGCCTATCCGTCTC
*FIZZ1*	F: ATCGTGGAGAATAAGGTCAAGGAAC
	R: CAAGCACACCCAGTAGCAGTC
*β-actin*	F: ACATCCGTAAAGACCTCTATGCC
	R: TACTCCTGCTTGCTGATCCAC

#### 2.5.7 Immunofluorescence detection of protein expression of macrophage polarization markers in lung tissue

After embedding, lung tissue samples were sectioned and deparaffinized to facilitate antigen retrieval. The tissue sections were blocked with 3% BSA for 30 min and incubated with an anti-CD80 antibody (1:2000) overnight at 4°C. Subsequently, the sections were incubated with the appropriate horseradish peroxidase-conjugated secondary antibody (1:500, green) at ambient temperature for 50 min, followed by incubation with tryptic soy agar in the dark at room temperature for 10 min. Following incubation, tissue sections were subjected to microwave heating for antigen retrieval. An anti-mannose receptor antibody (1:200) was then added, and the sections were incubated overnight at 4°C. Sections were then incubated with the appropriate CY3-conjugated secondary antibody (1:300, red) in the dark at ambient temperature for 50 min. Following incubation, DAPI (blue) was used for nuclear staining. Tissue autofluorescence was suppressed using an autofluorescence quencher, followed by the application of an anti-fluorescence quenching mounting medium. The specimens were examined under a fluorescence microscope. Image-Pro Plus 6.0 was employed to analyze the acquired images and determine the mean optical density value (IOD/area) for each field of view.

#### 2.5.8 Immunohistochemical detection of related protein expression in lung tissues

Following sectioning and dewaxing of paraffin-embedded lung tissues, antigen retrieval was performed using 1× citrate buffer (pH 6.0) as the repair solution, followed by endogenous enzyme blocking with 3% H_2_O_2_ solution. Tissue sections were incubated with 10% goat serum for blocking, then subjected to antibody incubation, DAB staining, and hematoxylin counterstaining. After dehydration and mounting, protein expression was detected using the PV-9000 universal two-step detection kit, with positive signals observed under an optical microscope. Five random microscopic fields were selected per sample section, and the acquired images were analyzed using Image-Pro Plus 6.0 software to calculate the mean optical density value (IOD/area) for each field.

### 2.6 Statistical analysis

Experimental data were processed and analyzed using SPSS 25.0 (SPSS Inc., Chicago, IL, USA). Data are expressed as mean ± standard deviation. For normally distributed data, comparisons among different groups were performed using one-way analysis of variance, with pairwise comparisons performed using the least significant difference test (for equal variances) or the Games–Howell test (for unequal variances). Non-parametric rank-sum tests were used for data that did not follow a normal distribution. The Kruskal–Wallis H test was initially used to assess overall differences, followed by the Mann–Whitney U test for pairwise group comparisons. A *P*-value < 0.05 was considered statistically significant.

## 3 Results

### 3.1 Identification of MXSGD components using UPLC-HRMS

**Figure 2:**
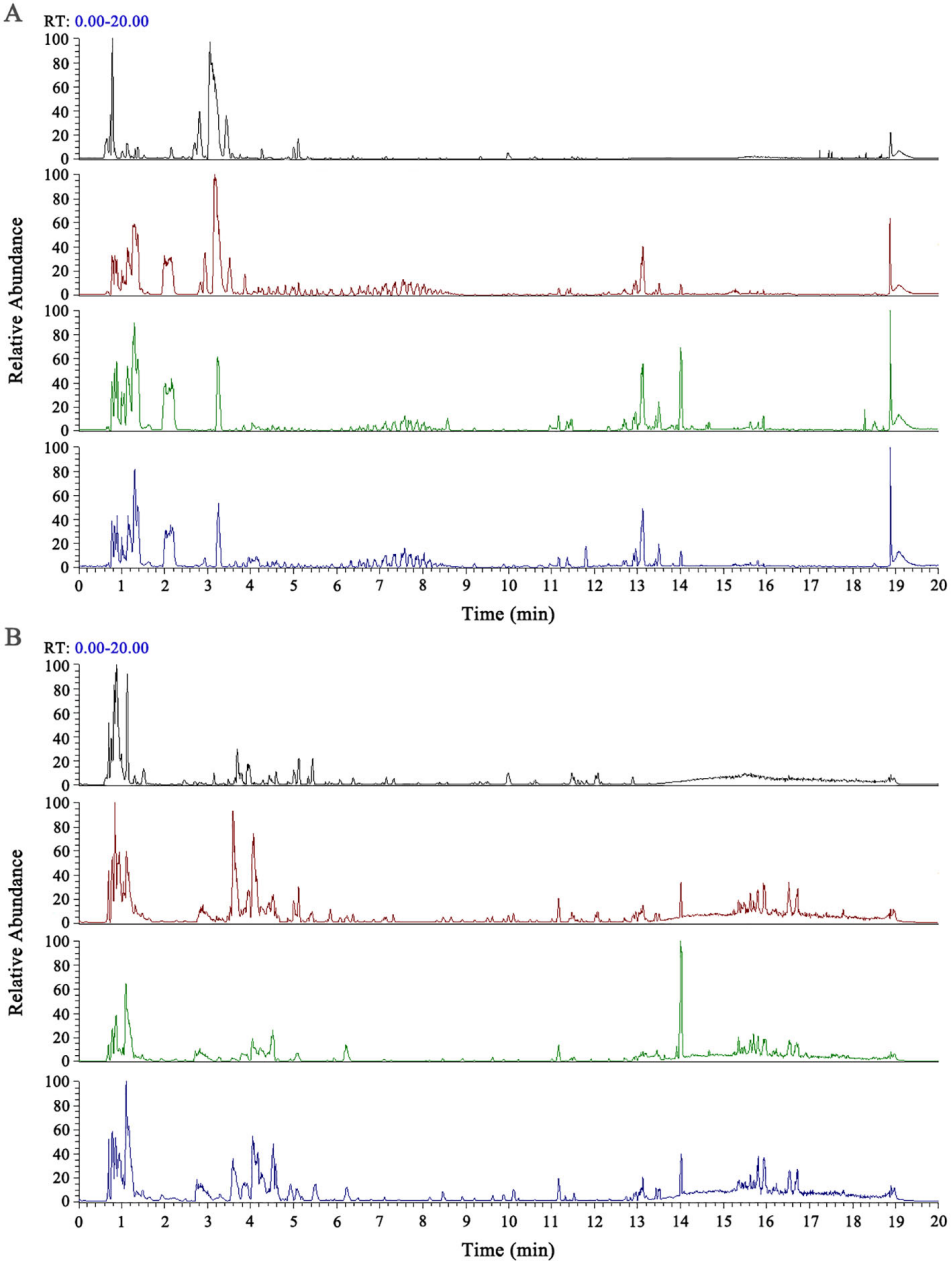
BPC graphs of each sample group. **(A)** BPC graphs of each group in positive ion mode. **(B)** BPC graphs of each group in negative ion mode. From top to bottom: MXSGD-PRE, CONTROL + MXSGD-PRE, CONTROL, MXSGD.

**Figure 3:**
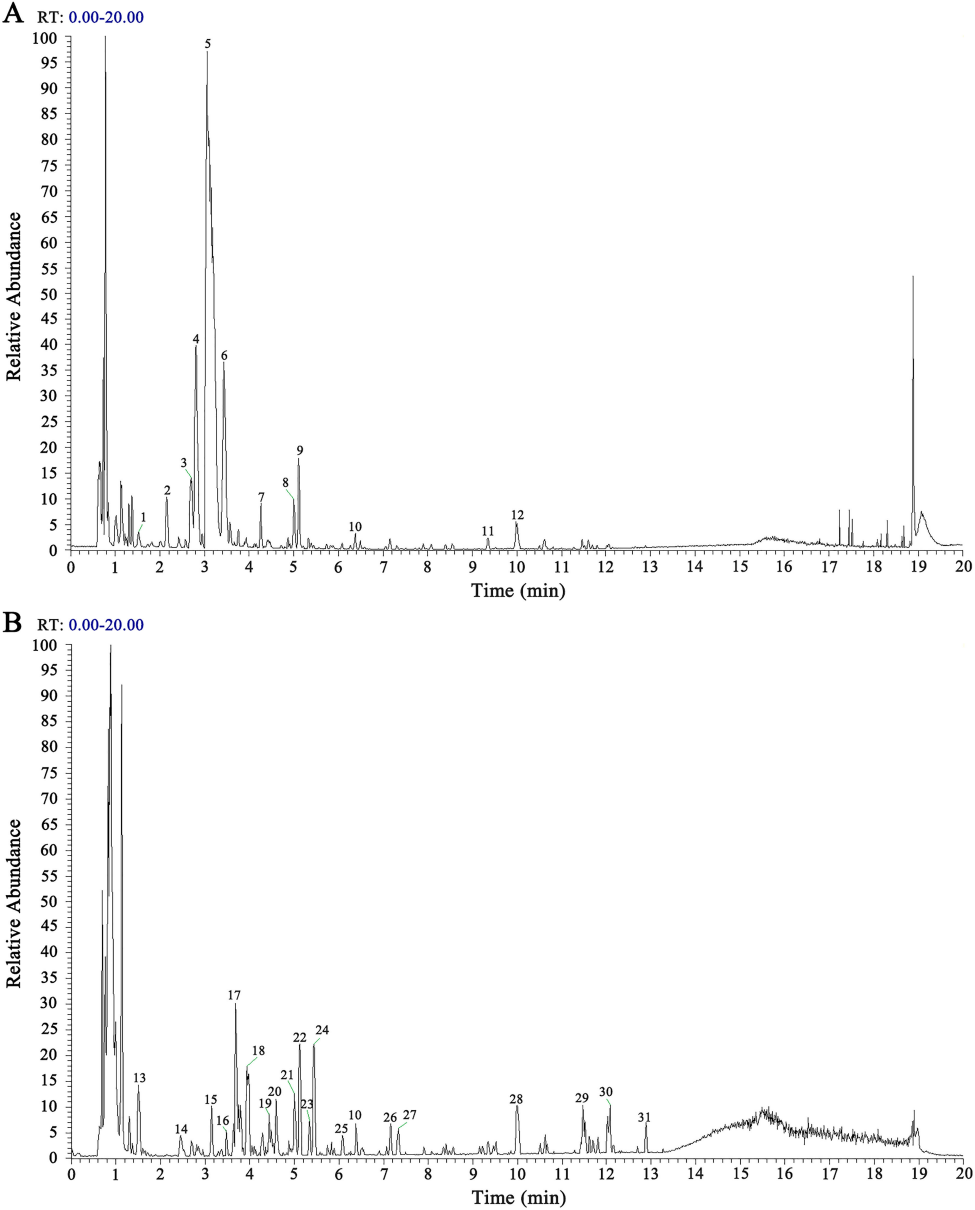
BPC graph of MXSGD-PRE (Marked Peaks). **(A)** BPC graph of MXSGD-PRE in positive ion mode. **(B)** BPC graph of MXSGD-PRE in negative ion mode.

Using UHPLC-HRMS, we collected data from the CONTROL, MXSGD, CONTROL + MXSGD-PRE, and MXSGD-PRE serum samples, and compared their positive and negative ion base peak chromatograms (BPCs) (**Figure 2**). In the positive and negative ion BPCs of MXSGD-PRE, high-abundance chromatographic peaks were validated based on peak shape and confirmed using the corresponding MS² spectra. Each identified peak was then sequentially labeled and marked in the positive and negative ion chromatograms (**Figure 3**).

We then transferred the resulting data to a local database of standard chromatograms relevant to TCM for MS² retrieval and comparison, identifying 1,297 chemical constituents in the blood following the administration of MXSGD. We identified 111 classes based on the ClassyFire classification method (22). Of them, the top 6 compounds included benzenes and their substituted derivatives (15.80%), carboxylic acids and their derivatives (22.12%), lipids (15.17%), flavonoids (15.42%), organic oxides (15.55%), and isoprenoids (15.93%).

Based on the BPCs of MXSGD-PRE (**Figure 3**), we conducted a preliminary identification of the typical base peaks, revealing 26 main active components of MXSGD, each with basic chemical formulas and clear definitions. Of them, 17 components were detected in the blood, 9 were not detected, and 5 remained unidentified (**Table 2**).

**Table 2:** Identification results of Characteristic Base Peaks of MXSGD from the BPC.

Peak number	No.	m/z	RT min	ppm	Compound name	Score	Class	Into Blood or None
1	M166T89 2	166.1228	1.49	1	Hordenine	0.9983	Benzene and substituted derivatives	None
2	M166T129 1	166.0861	2.15	17.6	Phenylalanine	0.9997	Carboxylic acids and derivatives	None
3	M134T175 3	134.0965	2.76	0.6	Phenylalanine,.alpha.-methyl-	0.9953	Phenylpropanoic acids	Into Blood
4	M152T175 2	152.1069	2.91	0.5	(+/-)-Norephedrine	0.9974	Benzene and substituted derivatives	Into Blood
5	M166T194 2	166.1227	3.24	0.1	DL-Ephedrine	0.9983	Benzene and substituted derivatives	Into Blood
6	M180T211	180.1382	3.51	0	N-Methylephedrine	0.9832	Benzene and substituted derivatives	Into Blood
7	M192T257	192.0655	4.28	0.4	4-Keto-8-methoxy-1H-quinoline-2-carboxylic acid	0.9981	Quinolines and derivatives	None
8	M551T300	551.1761	5.01	NA	NA	NA	NA	Into Blood
9	M257T307 5	257.0804	5.12	0.3	Liquiritigenin	0.9996	Flavonoids	Into Blood
10	M419T383 3	419.1335	6.38	1	Liquiritin	0.9947	Flavonoids	None
11	M839T561	839.4059	9.36	0.3	Licoricesaponin g2	0.9237	Prenol lipids	Into Blood
12	M453T600 5	453.3363	10.00	0	18.beta.-Glycyrrhetinic acid	0.9873	Prenol lipids	Into Blood
13	M173T93	173.0084	1.54	3.1	Isocitric acid	0.9985	Carboxylic acids and derivatives	None
14	M520T147 1	520.1679	2.45	1.4	2-((6-O-.beta.-D-Glucopyranosyl-.beta.-D-glucopyranosyl)oxy)-2-phenylacetamide	0.9033	Fatty Acyls	Into Blood
15	M951T187	951.3007	3.11	2.2	Physanguloside A	0.8615	Organooxygen compounds	None
16	M293T208 4	293.1240	3.46	NA	NA	NA	NA	Into Blood
17	M165T220 4	165.0555	3.67	3.2	Benzenepropanoic acid, 4-hydroxy-	0.994	Phenylpropanoic acids	Into Blood
18	M502T237 2	502.1575	3.94	0.9	((6-O-Hexopyranosylhexopyranosyl)oxy)(phenyl)acetonitrile	0.9311	Organooxygen compounds	Into Blood
19	M379T267 2	379.1979	4.44	17.1	Clausarin	0.8776	Coumarins and derivatives	Into Blood
20	M340T275 5	340.1040	4.58	0.5	(R)-Prunasin	0.9854	Organooxygen compounds	Into Blood
21	M549T301	549.1624	5.01	1.1	Liguiritigenin-7-O-beta-D-apiosyl-4’-O-beta-D-glucoside	0.9792	Flavonoids	None
22	M417T306 6	417.1196	5.10	1.9	Isoliquiritin	0.9862	Flavonoids	Into Blood
23	M245T319 5	245.0932	5.32	0.3	N-Acetyl-D-tryptophan	0.963	Carboxylic acids and derivatives	Into Blood
24	M463T327	463.0890	5.44	1.4	Spireoside	0.9979	Flavonoids	Into Blood
25	M187T364 3	187.0972	6.07	1.7	Azelaic acid	0.991	Fatty Acyls	None
10	M417T383 4	417.1197	6.38	1.5	Liquiritin	0.8948	Flavonoids	None
26	M255T429 5	255.0665	7.15	1.7	Isoliquiritigen	0.987	Linear 1 3 **-** diarylpropanoids	Into Blood
27	M361T439 2	361.1874	7.32	NA	NA	NA	NA	Into Blood
28	M821T600 5	821.3982	10.00	2.9	Glycyrrhizinate dipotassium	0.9847	Prenol lipids	Into Blood
29	M367T688 3	367.1191	11.47	1.1	Glycycoumarin	0.992	Isoflavonoids	None
30	M353T724 3	353.1035	12.06	NA	NA	NA	NA	Into Blood
31	M351T773 4	351.0879	12.89	NA	NA	NA	NA	Into Blood

### 3.2 Network pharmacology analysis of MXSGD

#### 3.2.1 Prediction of potential targets for the treatment of ALI based on the blood-absorbed components of MXSGD

Among the 1,297 components identified using UPLC-HRMS, we selected the blood-absorbed components of MXSGD with high average ion abundance values using the criteria of “into blood” and “mean ≥ e^8^.” We then intersected these components with the 17 main blood-absorbed components obtained from the BPCs, resulting in 56 core blood-absorbed components (**Table 3**). We used these 56 blood-absorbed components to retrieve component targets from the BATMAN-TCM, SwissTargetPrediction, and PharmMapper databases, removing duplicates and integrating 1,114 target points for the blood-absorbed components of MXSGD.

**Table 3:** Screening results of Core compounds that enter the blood of MXSGD.

Number	Molecular Formula	m/z	RT min	ppm	Compound name	Score	Class	Mean
1	C_10_H_15_NO	166.1227	3.24	0.1	DL-Ephedrine	0.9983	Benzene and substituted derivatives	3.89E+10
2	C_10_H_15_NO	148.1120	3.23	0	(-)-Pseudoephedrine	0.9967	Benzene and substituted derivatives	3.50E+10
3	C_10_H_13_NO_2_	134.0965	2.76	0.6	Phenylalanine,.alpha.-methyl-	0.9953	Phenylpropanoic acids	9.93E+09
4	C_11_H_17_NO	180.1382	3.51	0	N-Methylephedrine	0.9832	Benzene and substituted derivatives	8.68E+09
5	C_9_H_13_NO	152.1069	2.91	0.5	(+/-)-Norephedrine	0.9974	Benzene and substituted derivatives	3.32E+09
6	C_15_H_12_O_4_	257.0804	5.12	0.3	Liquiritigenin	0.9996	Flavonoids	2.08E+09
7	C_9_H_9_NO_3_	178.0505	4.16	2.8	3-Pyridinebutanoic acid,.gamma.-oxo-	0.9975	Keto acids and derivatives	1.71E+09
8	C_7_H_16_N_2_O_2_	144.1020	1.04	0.5	L-.beta.-Homolysine	0.9968	Carboxylic acids and derivatives	1.21E+09
9	C_20_H_27_NO_11_	502.1575	3.94	0.9	((6-O-Hexopyranosylhexopyranosyl)oxy)(phenyl)acetonitrile	0.9311	Organooxygen compounds	1.20E+09
10	C_14_H_17_NO_6_	340.1040	4.58	0.5	(R)-Prunasin	0.9854	Organooxygen compounds	9.23E+08
11	C_21_H_22_O_9_	417.1196	5.10	1.9	Isoliquiritin	0.9862	Flavonoids	8.99E+08
12	C_30_H_46_O_4_	453.3363	10.00	0	18.beta.-Glycyrrhetinic acid	0.9873	Prenol lipids	8.78E+08
13	C_9_H_10_O_3_	165.0555	3.67	3.2	Benzenepropanoic acid, 4-hydroxy-	0.994	Phenylpropanoic acids	8.59E+08
14	C_7_H_16_O_3_	149.1171	4.08	0.1	1-(2-Methoxyethoxy)-2-methyl-2-propanol	0.772	Organooxygen compounds	8.49E+08
15	C_42_H_62_O_16_	823.4111	10.00	1	Glycyrrhizin	0.9988	Prenol lipids	6.63E+08
16	C_11_H_15_N_5_O_4_	136.0625	0.84	0.6	2’-O-Methyladenosine	0.9999	Purine nucleosides	6.25E+08
17	C_10_H_10_O_5_	209.0454	3.66	2.8	3-(3,4-dihydroxy-5-methoxyphenyl)prop-2-enoic acid	0.9923	Cinnamic acids and derivatives	5.64E+08
18	C_12_H_22_O_11_	377.0861	0.79	0.8	.alpha.,.beta.-Trehalose	0.9818	Organooxygen compounds	4.86E+08
19	C_42_H_60_K_2_O_16_	821.3982	10.00	2.9	Glycyrrhizinate dipotassium	0.9847	Prenol lipids	4.58E+08
20	C_17_H_26_O_10_	211.0942	5.90	11.2	Methyl (1S)-1-(.beta.-D-glucopyranosyloxy)-6-hydroxy-7-methyl-1,4a,5,6,7,7a-hexahydrocyclopenta[c]pyran-4-carboxylate	0.9992	Prenol lipids	4.48E+08
21	C_15_H_14_O_6_	291.0862	3.80	1	Epicatechin	0.9987	Flavonoids	4.36E+08
22	C_9_H_11_N	117.0702	2.91	1.8	trans-2-Phenylcyclopropylamine	0.9991	Organonitrogen compounds	4.30E+08
23	C_20_H_23_NO	134.0602	2.44	0.5	N-Cyclohexyl-2,2-diphenylacetamide	0.9982	Benzene and substituted derivatives	4.15E+08
24	C_10_H_17_NO_3_	100.0761	2.11	4	N-Boc-2-piperidone	0.9996	Piperidines	3.71E+08
25	C_7_H_12_O_5_	175.0607	3.73	2.6	alpha-Isopropylmalate	0.9983	Fatty Acyls	3.11E+08
26	C_42_H_62_O_17_	839.4059	9.36	0.3	Licoricesaponin g2	0.9237	Prenol lipids	2.99E+08
27	C_11_H_15_NO_2_	194.1172	5.85	0.2	2-Amino-2-methyl-4-phenylbutyric acid	0.9604	Carboxylic acids and derivatives	2.91E+08
28	C_27_H_30_O_14_	579.1711	4.88	0.4	(1S)-1,5-Anhydro-2-O-(6-deoxy-.alpha.-L-mannopyranosyl)-1-(5,7-dihydroxy-2-(4-hydroxyphenyl)-4-oxo-4H-chromen-6-yl)-D-glucitol	0.9521	Flavonoids	2.90E+08
29	C_10_H_12_O_3_	137.0237	6.49	5.5	Isopropyl m-hydroxybenzoate	0.9989	Benzene and substituted derivatives	2.64E+08
30	C_10_H_11_NO_2_S	106.0655	2.48	3.4	2-Phenylthiazolidine-4-carboxylic acid	0.9989	Carboxylic acids and derivatives	2.51E+08
31	C_14_H_14_N_2_O_5_	291.0975	5.33	0.3	N-Malonyltryptophan	0.8316	Carboxylic acids and derivatives	2.44E+08
32	C_26_H_28_O_14_	565.1555	4.47	0.4	4H-1-Benzopyran-4-one, 6-arabinopyranosyl-8-.beta.-D-glucopyranosyl-5,7-dihydroxy-2-(4-hydroxyphenyl)-	0.9452	Flavonoids	2.39E+08
33	C_12_H_20_O_2_	137.1325	5.40	0.1	Neryl acetate	0.9984	Fatty Acyls	2.37E+08
34	C_26_H_30_O_11_	355.1176	12.05	0.3	Phellamurin	0.9872	Flavonoids	2.26E+08
35	C_15_H_12_O_4_	255.0665	7.15	1.7	Isoliquiritigen	0.987	Linear 1 3 **-** diarylpropanoids	2.26E+08
36	C_6_H_11_NO_3_	128.0707	0.81	2.1	2-Amino-5-oxohexanoic acid	0.9693	Carboxylic acids and derivatives	2.18E+08
37	C_22_H_22_O_9_	431.1337	6.49	0.2	Ononin	0.9997	Isoflavonoids	2.16E+08
38	C_10_H_18_O	137.1325	4.45	0.7	(+)-Isomenthone	0.996	Prenol lipids	1.97E+08
39	C_20_H_29_NO_12_	520.1679	2.45	1.4	2-((6-O-.beta.-D-Glucopyranosyl-.beta.-D-glucopyranosyl)oxy)-2-phenylacetamide	0.9033	Fatty Acyls	1.87E+08
40	C_13_H_14_N_2_O_3_	245.0932	5.32	0.3	N-Acetyl-D-tryptophan	0.963	Carboxylic acids and derivatives	1.82E+08
41	C_11_H_14_O_4_	193.0861	3.71	1.1	Sinapyl alcohol	0.9881	Phenols	1.75E+08
42	C_9_H_10_O_3_	165.0552	5.20	3.3	DL-3-Phenyllactic acid	0.9342	Phenylpropanoic acids	1.69E+08
43	C_11_H_11_NO_3_	131.0490	4.16	0.8	Cinnamoylglycine	0.9993	Carboxylic acids and derivatives	1.60E+08
44	C_15_H_21_NO_7_	328.1390	1.99	0.9	N-Fructosyl phenylalanine	0.8725	Carboxylic acids and derivatives	1.60E+08
45	C_26_H_30_O_13_	551.1762	6.09	0.4	4-(7-Hydroxy-4-oxo-3,4-dihydro-2H-chromen-2-yl)phenyl 2-O-(3,4-dihydroxy-4-(hydroxymethyl)tetrahydrofuran-2-yl)hexopyranoside	0.9819	Flavonoids	1.48E+08
46	C_30_H_44_O_4_	469.3312	8.56	0.3	Glabrolide	0.9808	Prenol lipids	1.41E+08
47	C_8_H_17_NO_2_	160.1332	0.84	0.3	5-aminovaleric acid betaine	0.9892	Fatty Acyls	1.40E+08
48	C_18_H_24_O_12_	433.1340	3.93	0.6	Licoagroside B	0.9994	Saccharolipids	1.33E+08
49	C_18_H_33_C_l_N_2_O_5_S	425.1780	8.09	21.9	Clindamycin	0.8949	Carboxylic acids and derivatives	1.31E+08
50	C_27_H_30_O_15_	595.1660	4.14	1.7	Vicenin-2	0.9789	Flavonoids	1.14E+08
51	C_21_H_20_O_6_	369.1334	10.50	0.3	Icaritin	0.9458	Flavonoids	1.14E+08
52	C_13_H_13_NO_4_	218.0457	4.26	0.8	Ethyl 4-hydroxy-7-methoxy-3-quinolinecarboxylate	0.9906	Quinolines and derivatives	1.04E+08
53	C_13_H_16_O_9_	315.0726	2.79	8.5	Benzoic acid + 2O, O-Hex	0.8777	Organooxygen compounds	1.04E+08
54	C_24_H_28_O_4_	379.1979	4.44	17.1	Clausarin	0.8776	Coumarins and derivatives	1.03E+08
55	C_7_H_10_O_4_	113.0601	5.71	2.2	Succinylacetone	0.9809	Keto acids and derivatives	1.02E+08
56	C_15_H_12_O_5_	273.0749	5.84	0.5	Naringenin chalcone	0.9978	Linear 1 3 **-** diarylpropanoids	1.01E+08

Following the consolidation and elimination of duplicates from all ALI targets received from the GeneCards, DisGeNET, and OMIM databases, we identified 3,037 genes associated with ALI. These genes were then intersected with 1,114 target points of the blood-absorbed components of MXSGD, revealing 338 overlapping targets.

#### 3.2.2 Construction of the C–T network

**Figure 4:**
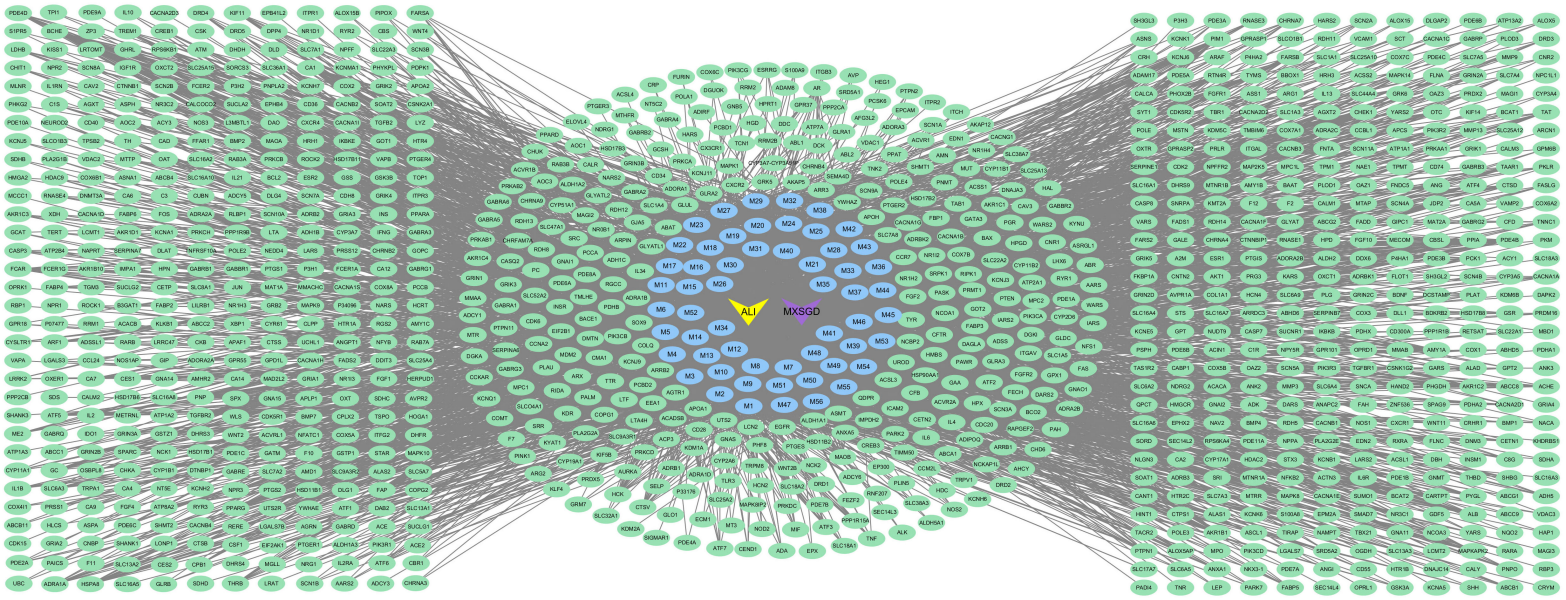
The C-T network of MXSGD. Purple nodes refer to MXSGD. Green nodes refer to the potential targets. Blue nodes refer to the 56 constituents absorbed into blood contained in MXSGD (M1-M56 represents the active component of MXSGD with serial numbers 1–56 in **Table 3**).

We generated the C–T network using Cytoscape software (**Figure 4**) and analyzed it using the Network Analyzer plugin. A higher degree indicates that a node is connected to more nodes, reflecting a more significant regulatory role in the overall network. The network contained 1,172 nodes, including one Chinese herbal formula, 56 blood-absorbed components, 1,114 action targets, and 1 disease, as well as 4,619 edges.

#### 3.2.3 Construction and analysis of the PPI network

**Figure 5:**
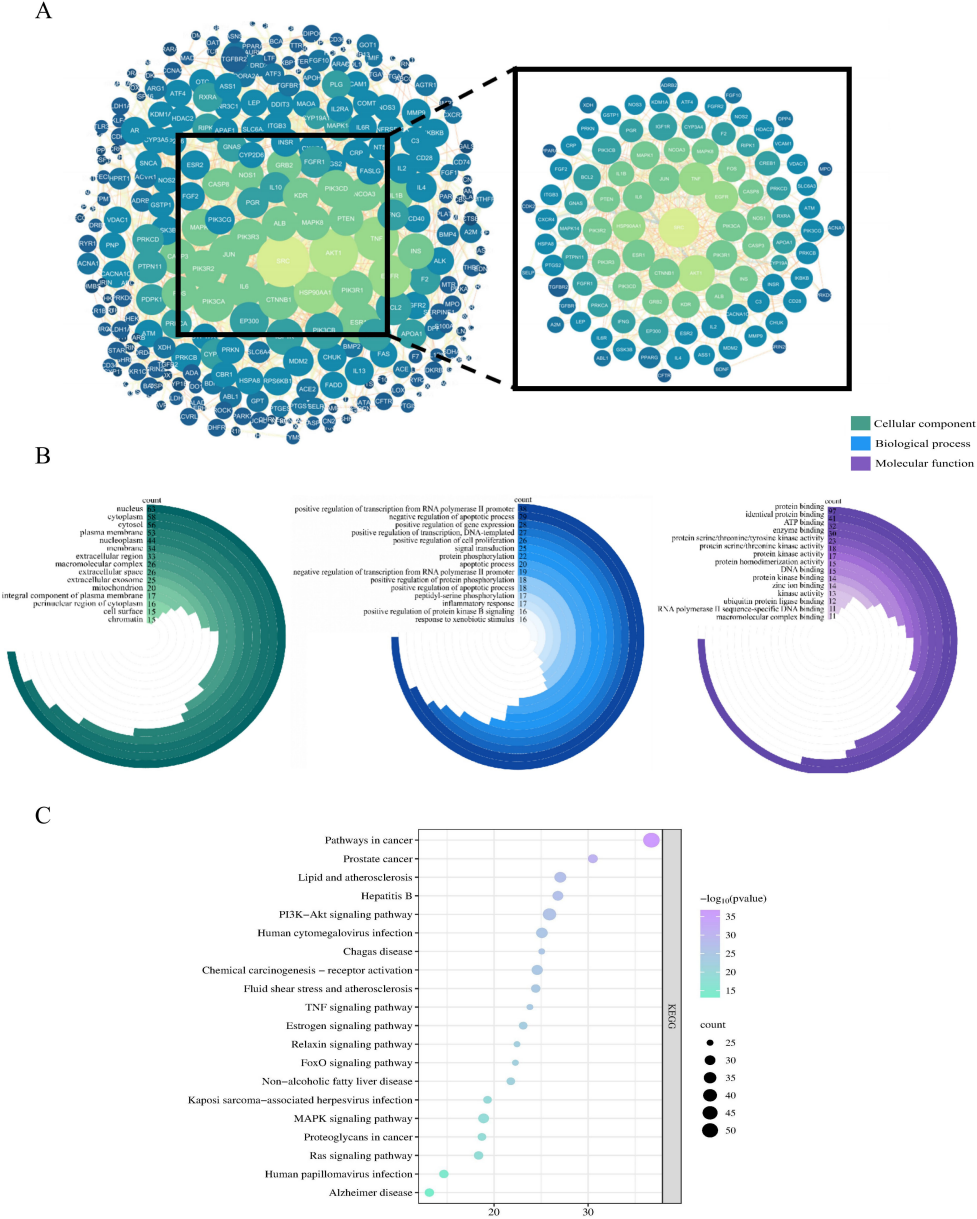
PPI and Enrichment analysis of the key targets. **(A)** Protein-protein interaction (PPI) network of potential targets for MXSGD treatment of ALI. **(B)** Top 15 significantly enriched terms in biological processes, molecular functions, and cellular components. **(C)** Top 20 significantly enriched terms in KEGG pathways.

We imported the 338 selected intersection targets into the STRING database to determine the interaction relationships between proteins. Each circle represents a protein node participating in the interactions, and each line represents the interactions between targets. Larger circles indicate higher degree values, and thicker lines indicate higher binding scores. The resulting PPI network contained 304 nodes, with 34 targets not participating in the interactions, and 916 interaction lines. The network yielded a median degree of 4, a median betweenness of 182.669, and an average closeness of 0.051 resulting in 99 core targets (**Figure 5A**).

Among these key targets, the primary ones included tumor necrosis factor (TNF) and interleukin (IL)-1β, which are associated with inflammation; HSP90AA1 and SRC, which are associated with cell proliferation and carcinogenesis; epidermal growth factor receptor (EGFR), which is linked to vascular endothelium; and AKT, Phosphoinositide-3-kinase regulatory subunit 1 (PIK3R1), and phosphatidylinositol 4,5-bisphosphate 3-kinase catalytic subunit alpha (PIK3CA), which are critical components of the PI3K/AKT pathway. This result indicates that MXSGD influences the regulation of inflammatory responses, oxidative stress, autophagy, apoptosis, and vascular function.

Further analysis of these key targets revealed that many of them were closely associated with macrophage polarization. For example, inflammation-related targets such as TNF, interferon gamma (IFNG), and prostaglandin-endoperoxide synthase 2 (PTGS2) are involved in the macrophage polarization process. Several signaling pathways associated with key targets were also identified as critical pathways regulating macrophage polarization, including AKT1, PIK3R1, PIK3CA, Phosphatase and tensin homolog (PTEN), mitogen-activated protein kinase 1 (MAPK1), MAPK14, inhibitor of nuclear factor kappa-b kinase subunit beta (IKBKB), and conserved helix-loop-helix ubiquitous kinase (CHUK). These key targets included transcription factors related to macrophage polarization, such as peroxisome proliferator-activated receptor gamma (PPARG) and JUN/FOS (AP-1 complex), and markers associated with macrophage polarization, including nitric oxide synthase 2 (NOS2).

#### 3.2.4 GO enrichment analysis

We performed GO analysis of the principal targets of the blood-absorbed components of MXSGD for the treatment of ALI using DAVID 2021, with *P* < 0.05 set as the criterion, revealing a total of 915 GO items. The GO enrichment analysis is primarily categorized into three domains: biological processes (BP), cellular components (CC), and molecular functions (MF). The BP-related entries were the most abundant, totaling 696, and predominantly related to the positive regulation of gene expression, signal transduction, protein phosphorylation, negative regulation of apoptotic process, positive regulation of transcription and DNA-templated, and immunological responses. The CC-related entries totaled 81, mainly involving the cytosol, cytoplasm, nucleus, and nucleoplasm. The MF-related entries included 138 entries, primarily focusing on protein binding, ATP binding, identical protein binding, enzyme binding, and protein kinase activity and binding. These GO entries were sorted by count values, and the top 15 entries for BP, CC, and MF were selected and visualized using a bubble chart (**Figure 5B**).

#### 3.2.5 KEGG pathway analysis

We conducted KEGG analysis of the potential treatment targets of ALI by MXSGD using DAVID (2021; with *P* < 0.05 as the criterion), revealing 169 enriched KEGG pathways. The top 20 pathways are displayed in a bubble chart in **Figure 5C**.

Most of the targets were enriched in signaling pathways associated with cancer, the PI3K/AKT signaling pathway, lipid metabolism, atherosclerosis, human cytomegalovirus infection, the MAPK signaling pathway, and Alzheimer’s disease.

#### 3.2.6 Construction of the CTP network

**Figure 6:**
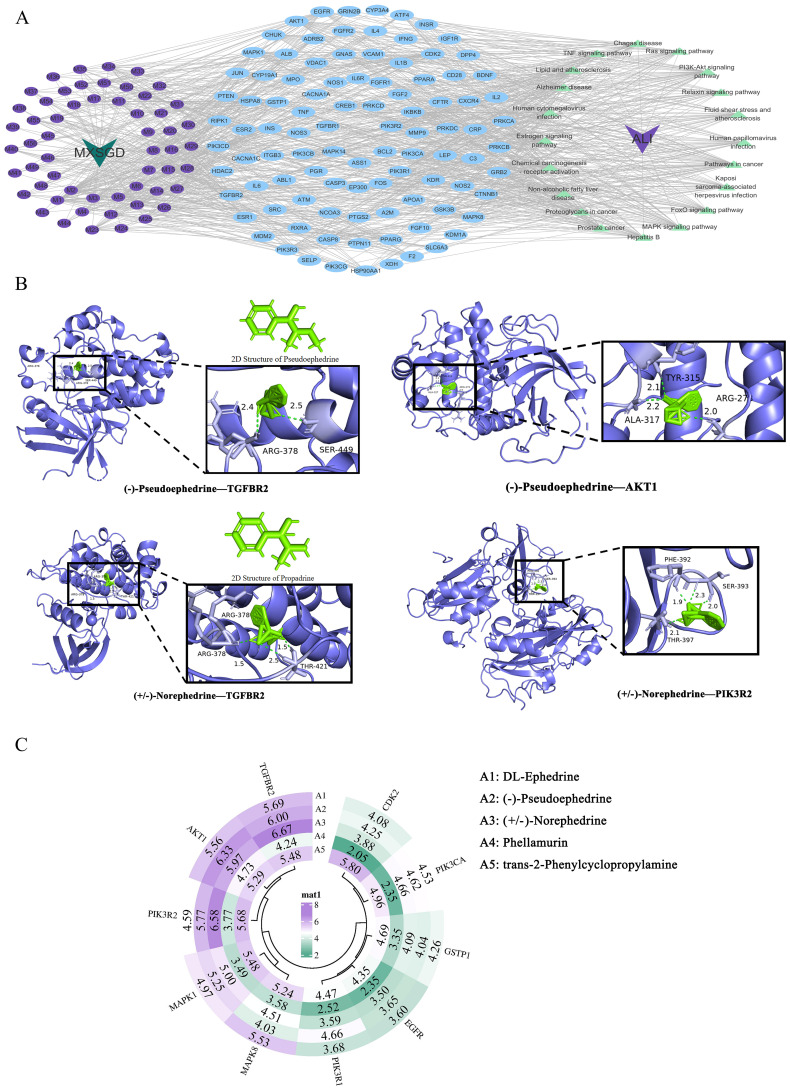
CTP network and molecular docking results. **(A)** CTP network of MXSGD. Purple nodes refer to MXSGD. Blue nodes refer to the 99 key targets. Green nodes on the right refer to the signal pathways. Purple nodes on the left refer to the 56 constituents absorbed into blood contained in MXSGD (M1-M56 represents the active component of MXSGD with serial numbers 1–56 in **Table 3**). **(B)** Representative docking complex of key targets and compounds. **(C)** Pie chart of docking scores of 10 key targets combining to 5 active compounds in MXSGD. Binding energy values in **Figure 6C** are negative.

We constructed the CTP network using Cytoscape software (**Figure 6A**) and analyzed it using the Network Analyzer plugin. Higher node degree values indicate a greater number of connected nodes, reflecting enhanced regulatory function within the entire network. The network contained 176 nodes, including 1 Chinese herbal formula, 56 blood-absorbed components, 99 key targets, 20 signaling pathways, and 1 disease, as well as 1,124 edges. These findings highlight the characteristic therapeutic approach of TCM formulas, which act through a multi-component, multi-target, multi-pathway mechanism.

#### 3.2.7 Molecular docking

We selected degree-ranking compounds in the CTP network, including DL-ephedrine, (-)-pseudoephedrine, (+/-)-norephedrine, phellamurin, and trans-2-phenylcyclopropylamine, for molecular docking validation with EGFR, CDK2, MAPK1, GSTP1, MAPK8, TGFBR2, PIK3R2, AKT1, PIK3R1, and PIK3CA. The minimum binding energies for each protein-ligand complex were calculated. A binding energy below zero indicates spontaneous binding between ligand molecules and receptor proteins, and a binding energy below –1.2 kcal/mol suggests strong binding activity between the molecules. The smaller the binding energy, the stronger the binding ability, indicating better docking.

The four docking results with the best binding activity are visualized in **Figure 6C**. The four pharmacologically relevant blood-absorbed components exhibited good binding to target proteins. Specifically, the binding energies of (-)-pseudoephedrine and TGFBR2, (-)-pseudoephedrine and AKT1, (+/-)-norephedrine and TGFBR2, (+/-)-norephedrine and PIK3R2, and DL-ephedrine and TGFBR2 in the blood-absorbed components were all less than –1.2 kcal/mol. Among these, (+/-)-norephedrine exhibited the lowest binding energy with TGFBR2, followed by (+/-)-norephedrine with PIK3R2, indicating that (+/-)-norephedrine exhibited the strongest binding activity with TGFBR2 and PIK3R2. The docking results depicting the best visual binding activity are presented in **Figure 6B**.

### 3.3 Effects of MXSGD on IAV-induced lung injury mouse models

#### 3.3.1 Protective effects of MXSGD on IAV-induced ALI

**Figure 7:**
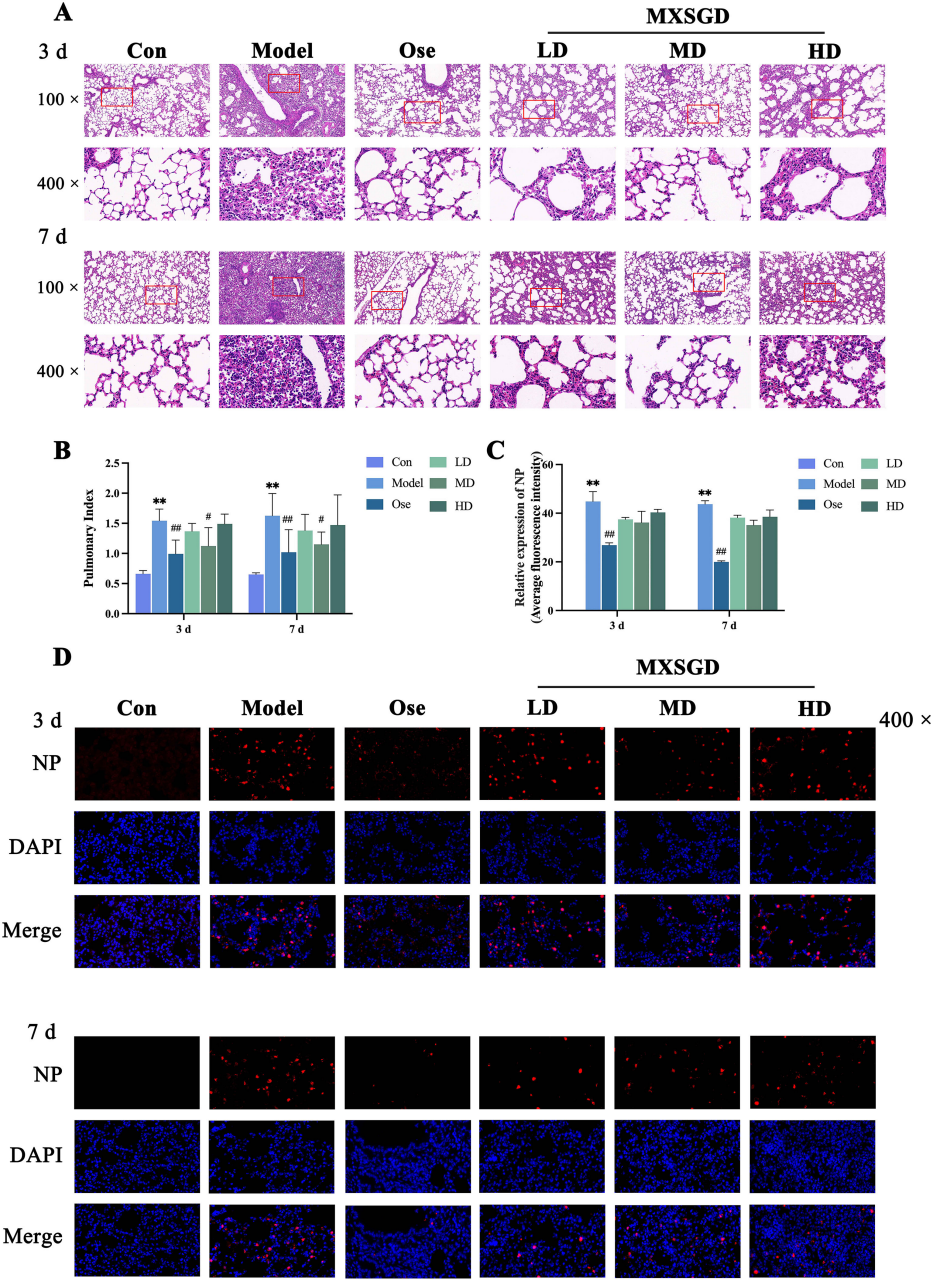
Effects of MXSGD on lung tissue induced by IAV. **(A)** Pathological changes in lung tissue of mice in each group (HE). **(B)** Comparison of lung index of mice in each group ( $\bar{x}$ ± *s*, *n* = 8). **(C)** Average fluorescence intensity of NP in each group ( $\bar{x}$ ± *s*, *n* = 3, × 400). **(D)** Immunofluorescence staining of NP in lung tissue. Compared to the control group, *P < 0.05, **P < 0.01; compared to the model group, ^#^ *P* < 0.05, ^##^ *P* < 0.01.

We then investigated the effects of MXSGD *in vivo* using IAV-induced lung injury mouse models. Histological analysis revealed intact alveolar morphology and septal structure in control mice. However, the model control group exhibited significant histopathological changes in lung tissue (**Figure 7A**), characterized by pulmonary congestion and edema, extensive infiltration of lymphocytes and macrophages, and large necrotic solid lesions. Compared to that in the model group, lung tissue histopathological damage in the MXSGD treatment groups significantly improved on days 3 and 7 post-treatment, as evidenced by reduced congestion and edema, decreased inflammatory cell infiltration, and a smaller area of necrotic solid lesions. These improvements were the most pronounced in the oseltamivir (positive treatment group) and medium-dose MXSGD groups, which exhibited clear alveolar outlines and reduced inflammatory cell infiltration.

The lung index data (**Figure 7B**) indicated that the model group exhibited a significant increase in the lung index on both days 3 and 7 post-IAV infection. In contrast, subsequent treatment with MXSGD led to a reduction in the lung index across all treatment groups to varying extents. Specifically, the lung index in the model group significantly increased compared to that in the control group (*P* < 0.01). Moreover, both the oseltamivir and medium-dose MXSGD groups exhibited a significant reduction in the lung index compared to the model group (*P* < 0.01 or *P* < 0.05).

#### 3.3.2 MXSGD decreases IAV levels in lung tissue

Our immunofluorescence analysis (**Figures 7C, D**) revealed significantly increased nuclear protein (NP) levels in lung tissues in the model groups on days 3 and 7 post-IAV infection relative to those in the control group *(P* < 0.01). However, following therapeutic intervention with MXSGD, the NP levels in all treatment groups decreased to varying degrees. On days 3 and 7 post-infection, the oseltamivir group demonstrated the significant decrease compared to the model group (*P* < 0.01).

### 3.4 Validation of the potential mechanisms underlying the therapeutic effects of MXSGD in lung injury models induced by IAV

The pathophysiological mechanism of ALI involves numerous cells and effector cells, among which macrophages are the most important cells in the innate immune system. A growing body of evidence indicates that, beyond their role in immune defense, pulmonary macrophages also play an important role as effector cells in regulating the local inflammatory microenvironment and inflammatory response in lung tissue (23). Macrophages exhibit high plasticity and heterogeneity, polarizing into classical activated M1 macrophages and alternatively activated M2 macrophages depending on the microenvironment. These two subtypes have opposing functions: M1 macrophages promote inflammation, whereas M2 macrophages suppress it (24).

Our network pharmacology analysis revealed that the blood-absorbed components of MXSGD ameliorate ALI through key target proteins and signaling pathways significantly associated with macrophage polarization. Notably, among the 99 identified key targets, core mediators such as TNF, IL6, IL1B, and IFNG play dual roles as central inflammatory cytokines and key regulators of macrophage polarization. Furthermore, several identified genes encode signature and effector molecules critical for macrophage polarization. For example, *Ptgs2* encodes cyclooxygenase-2 (COX-2), while *Nos2* produces inducible nitric oxide synthase (iNOS). Additionally, these key targets encompass essential transcription regulators such as PPARG, JUN, and FOS.

#### 3.4.1 MXSGD induces changes in pro-inflammatory cytokine levels in serum

**Figure 8:**
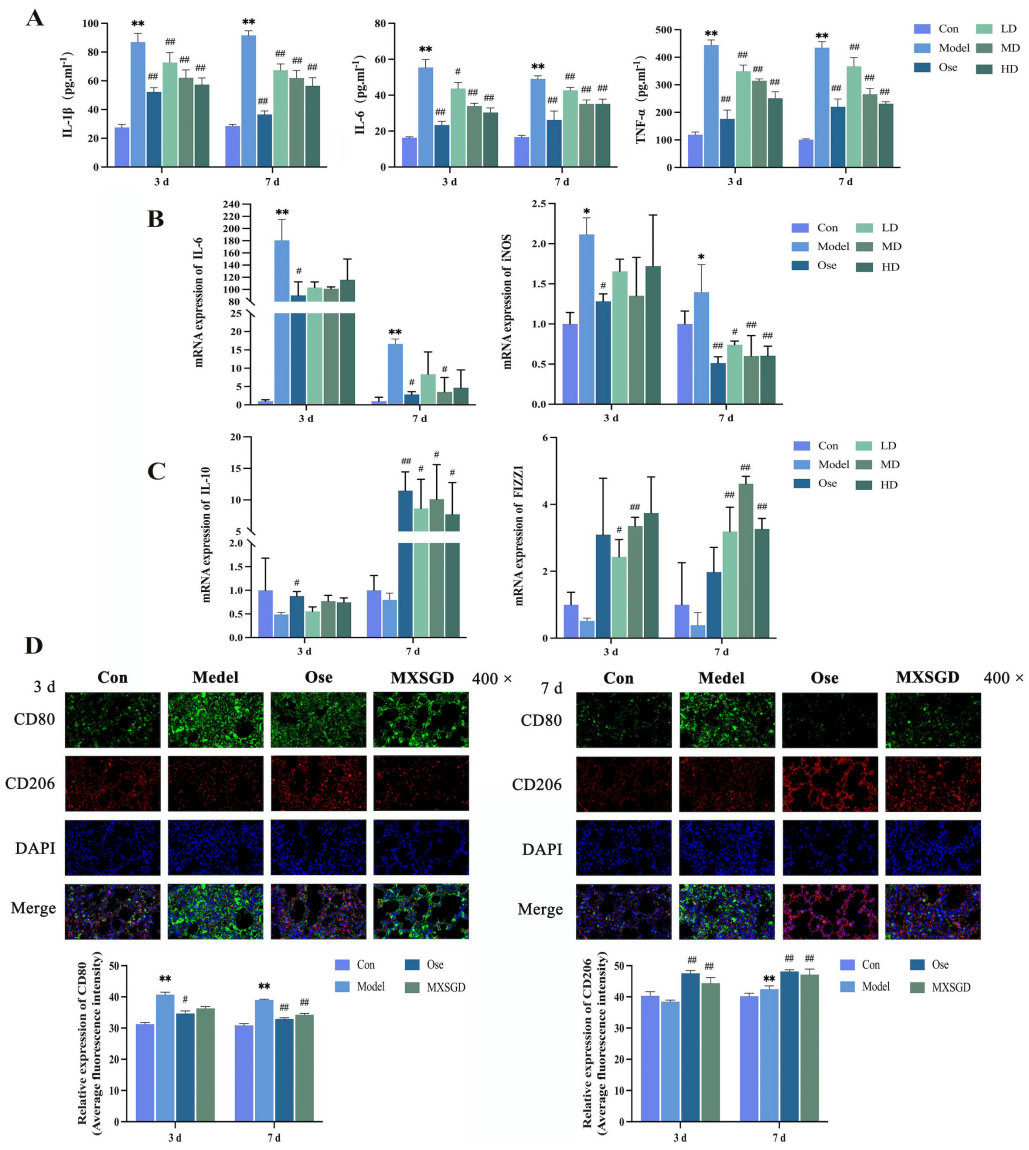
Verification of the potential mechanism of MXSGD in a lung injury model induced by influenza. **(A)** Effects of MXSGD on the levels of pro-inflammatory cytokines in serum ( $\bar{x}$ ± *s*, *n* = 5). **(B)** The mRNA Expression of IL-6 and iNOS in lung tissue ( $\bar{x}$ ± *s*, *n* = 4). **(C)** The mRNA Expression of IL-10 and FIZZ in lung tissue ( $\bar{x}$ ± *s*, *n* = 4). **(D)** Immunofluorescence staining of macrophage polarization markers CD80 and CD206 in lung tissue ( $\bar{x}$ ± *s*, *n* = 4). Compared to the control group, *P < 0.05, **P < 0.01; compared to the model group, ^#^ *P* < 0.05, ^##^ *P* < 0.01.

Based on the above findings, we subsequently investigated whether MXSGD confers protection against IAV-induced ALI by modulating macrophage polarization. To this end, we measured the levels of inflammatory cytokines in mouse sera. The results are presented in **Figure 8A**.

Following IAV infection, the model group exhibited a significant increase in IL-1β, IL-6, and TNF-α levels on days 3 and 7 (*P* < 0.01). However, following treatment with oseltamivir and MXSGD, the serum levels of IL-1β, IL-6, and TNF-α significantly decreased in all drug-treated mouse groups compared to the model group at both time points (*P* < 0.01 or *P* < 0.05). These results demonstrate that treatment with MXSGD reduces the serum levels of pro-inflammatory cytokines induced by IAV in mice.

#### 3.4.2 MXSGD alters the mRNA expression of *Il*-6, *iNos*, *Il-10*, and *Fizz* in lung tissue

We then analyzed the gene expression of various molecules in the lung tissue of mice from different groups (**Figure 8B**). They included IL-6, a classic pro-inflammatory cytokine; iNOS, an enzyme induced by inflammatory signaling, which synergizes with pro-inflammatory cytokines to amplify inflammatory responses and serves as a marker of M1 macrophages; IL-10, a classic anti-inflammatory cytokine; and FIZZ1, a secretory protein involved in inflammatory regulation and a marker of M2 macrophages.

Compared to that in the control group, *Il-6* expression was significantly increased in the model group on both days 3 and 7 post-IAV intervention (*P* < 0.01). Compared to that in the model group, the mRNA expression of *Il-6* was decreased in the oseltamivir group and all MXSGD dose groups. On day 3, the most pronounced reduction occurred in the oseltamivir group (*P* < 0.05). On day 7, we observed significant decreases in the oseltamivir group and the medium-dose MXSGD group (*P* < 0.05; **Figure 8B**).

Compared to that in the control group, *iNos* expression was significantly increased in the model group on both days 3 and 7 post-IAV infection (*P* < 0.05). All MXSGD -treated groups exhibited marked reductions in *iNos* expression compared to the model group. On day 3 post-infection, the most significant decrease occurred in the oseltamivir group (*P* < 0.05). On day 7, both the oseltamivir group and all MXSGD treatment groups demonstrated statistically significant reductions in *iNos* expression (*P* < 0.05 or *P* < 0.01; **Figure 8B**).

The expression of *Il-10* was downregulated in the model group on days 3 and 7 post-IAV infection compared to those in the control group; however, this decrease was not statistically significant. Compared to that in the model group, *Il-10* expression was upregulated in all drug-treated groups. Specifically, on day 3 post-infection, the most significant increase occurred in the oseltamivir group (*P* < 0.05). On day 7, *Il-10* expression was significantly upregulated in the oseltamivir group and all MXSGD treatment groups (*P* < 0.05 or *P* < 0.01; **Figure 8C**).

On days 3 and 7 post-IAV infection, *Fizz1* expression decreased in the model group compared to that in the control group, though without statistical significance. All drug-treated groups exhibited increased *Fizz1* expression compared to that in the model group. On day 3 post-infection, the low-dose and medium-dose MXSGD groups exhibited the most significant elevation (*P* < 0.05 or *P* < 0.01). On day 7, all MXSGD dose groups exhibited significant upregulation in *Fizz1* expression compared to the model group (*P* < 0.01; **Figure 8C**). These findings suggest that MXSGD can regulate the gene expression of macrophage polarization markers (*iNos* and *Fizz1*) in the lung tissue of IAV-infected mice.

#### 3.4.3 MXSGD affects macrophage polarization in lung tissue

Based on our preliminary experimental findings, and to further investigate the mechanistic relationship between MXSGD and macrophage polarization, we selected the following groups for subsequent analysis: control, model, oseltamivir, and MXSGD medium-dose groups. We subsequently characterized macrophage phenotypes in lung tissue using immunofluorescence double staining.

In terms of M1 macrophages, immunofluorescence revealed that the model group exhibited a significant increase in the expression of the M1 macrophage marker CD80 on both days 3 and 7 post-IAV infection, compared to the control group (*P* < 0.01; **Figure 8D**). Conversely, CD80 expression levels decreased to different extents in all MXSGD groups compared to those in the model group. On day 3 post-infection, the oseltamivir group exhibited a significant decrease in CD80 expression (*P* < 0.05). By day 7 post-infection, both the oseltamivir and MXSGD groups exhibited a significant decrease in CD80 expression (*P* < 0.01).

In terms of M2 macrophages, immunofluorescence revealed notable alterations in the M2 macrophage marker CD206. Compared to that in the control group, there were no notable alterations in CD206 levels in the model group on day 3 post-infection; however, on day 7, CD206 expression significantly increased (*P* < 0.01; **Figure 8D**). The oseltamivir and MXSGD groups exhibited a significant increase in CD206 levels on both days 3 and 7 compared to the model group (*P* < 0.01). This finding indicates that the macrophages were successfully repolarized from M1 to M2.

Overall, these findings suggest that MXSGD can mitigate the inflammatory response and pulmonary damage following influenza infection by modulating macrophage polarization.

#### 3.4.4 MXSGD modulates protein expression in the PI3K/AKT pathway

Macrophage polarization is regulated by multiple signaling pathways. In the present study, analysis of key targets revealed that several target-associated pathways critically govern this process, notably the PI3K/AKT, MAPK, and NF-κB pathways. Among them, the PI3K/AKT signaling cascade plays a central regulatory role in pulmonary injury pathologies. Thus, to validate these network pharmacology predictions, we performed IHC analysis of key proteins in the PI3K/AKT pathway on lung tissues from four experimental groups: control, model, oseltamivir, and MXSGD medium-dose groups.

**Figure 9:**
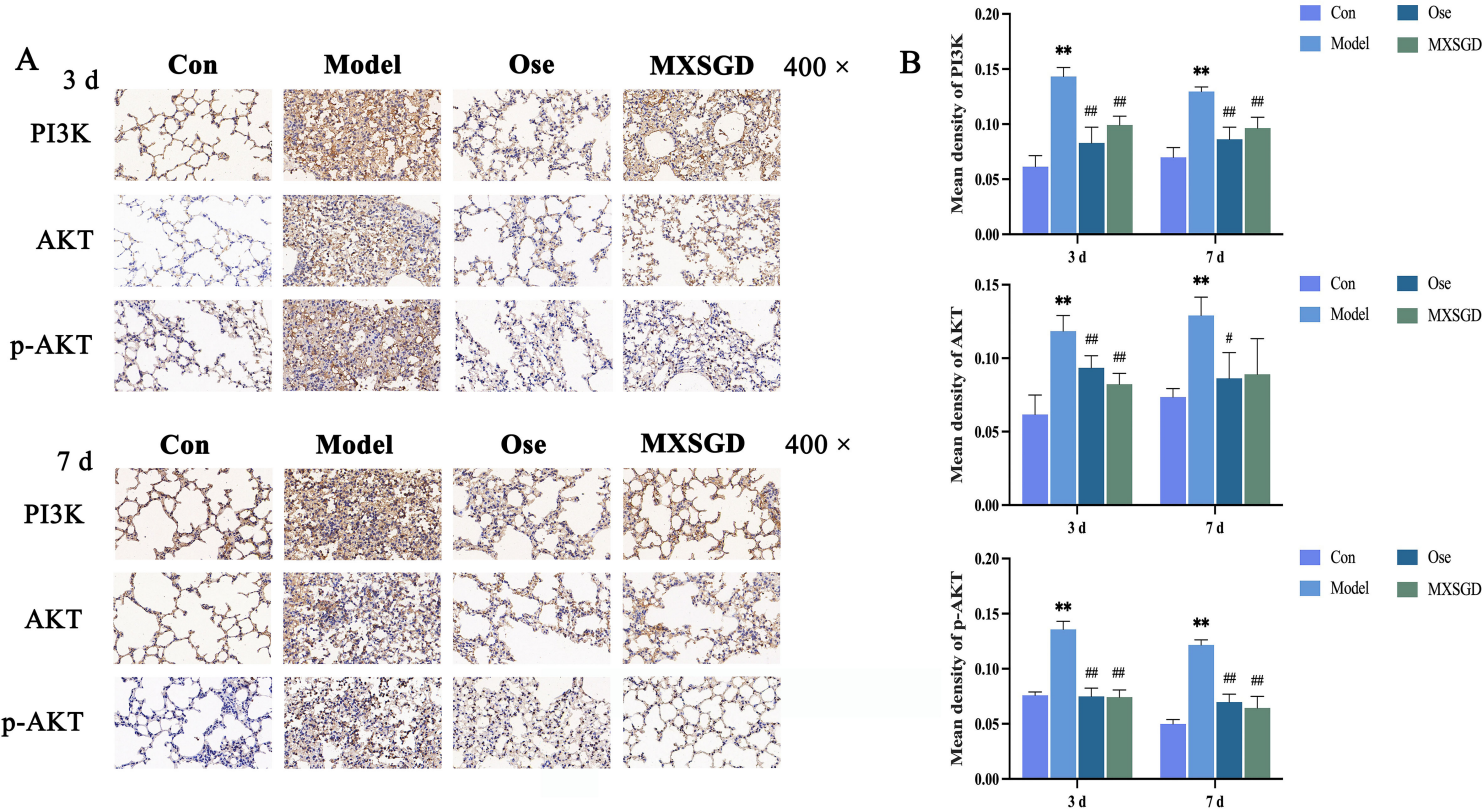
Expression of PI3K/AKT pathway-related proteins in lung tissues of mice across experimental groups. **(A)** Alterations in PI3K, AKT, and p-AKT Expression in Lung Tissues of Mice from Different Groups. **(B)** Comparison of Mean Optical Density (MOD) Values for PI3K, AKT, and p-AKT in Lung Tissues Across Groups ( $\bar{x}$ ± *s*, *n* = 5). Compared to the control group, *P < 0.05, **P < 0.01; compared to the model group, ^#^ *P* < 0.05, ^##^ *P* < 0.01.

Compared to that in the control group, PI3K protein expression in the model group was significantly upregulated in lung tissues on both days 3 and 7 following IAV infection (*P* < 0.01; **Figure 9**). However, both the oseltamivir and MXSGD groups exhibited a significant reduction in PI3K expression levels, compared to the model group (*P* < 0.01).

Similarly, AKT protein expression in the model group was significantly elevated on days 3 and 7 post-IAV infection compared to that in the control group (*P* < 0.01; **Figure 9**). However, the drug-treated groups exhibited reduced AKT expression compared to the model group. On day 3, AKT expression levels in both the oseltamivir and MXSGD groups were significantly lower than those in the model group (*P* < 0.01). By day 7, AKT expression significantly decreased in the oseltamivir group than that in the model group (*P* < 0.05).

We observed a consistent trend for phosphorylated AKT (p-AKT) expression (**Figure 9**). Specifically, the model group displayed a significant increase in p-AKT levels at both time points compared to the control group (*P*< 0.01), whereas therapeutic intervention with oseltamivir or MXSGD resulted in a significant decrease in p-AKT expression, compared to the model group (*P* < 0.01).

## 4 Discussion

IAV is a prevalent cause of respiratory infections in humans, and severe IAV-induced lung infection can lead to ALI by inducing macrophages to secrete inflammatory mediators. Notably, ALI is a significant contributor to mortality associated with IAV infection (25). Although lung-protective ventilation and neuromuscular blockers are effective in treating ALI, the mortality rate remains approximately 40%, primarily owing to multiple organ failure induced by inflammatory mediators (26–28). Consequently, mitigating the intense acute inflammatory response in individuals with ALI or ARDS is crucial for improving outcomes. MXSGD is a TCM formula used to treat influenza infections; however, the specific components mediating the therapeutic actions of MXSGD and its underlying mechanisms remain unknown. Therefore, in the present study, we used UPLC-HRMS to determine the blood-absorbed components of MXSGD and investigate the potential mechanisms underlying the therapeutic effects of MXSGD in ALI using network pharmacology and molecular docking techniques. Our findings demonstrated that MXSGD alleviates ALI through mechanisms involving viral inhibition, regulation of the inflammatory response, immune modulation, apoptosis, and oxidative stress.

In the present study, we analyzed blood-absorbed components, key targets, and signaling pathways and identified DL-ephedrine, pseudoephedrine, norephedrine, phellamurin, and 18β-glycyrrhetinic acid (18β-GA) as the components with the highest degree values in the network, indicating their central roles in the therapeutic effect. Ephedra is widely used in TCM to treat diseases such as bronchial asthma, fever, cough, and colds (29). DL-ephedrine is an isomer of the alkaloid ephedrine derived from ephedra. Ephedrine can effectively facilitate bronchial dilatation, mitigate inflammatory reactions, and inhibit cough reflexes. It can also suppress airway hyperreactivity in asthmatic mice by modulating the TGF-β1/Smads and TGF-β1/NF-κB signaling pathways, thereby enhancing airway remodeling and diminishing lung inflammation (30, 31). In rats with knee osteoarthritis, ephedrine can suppress the NF-κB signaling pathway by activating the AMPK pathway, thus inhibiting inflammatory responses and ameliorating cartilage damage (32). Pseudoephedrine is a significant constituent of ephedra, and compared to ephedrine, it exhibits a diminished vasoconstrictive action and a reduced influence on the central nervous system (33). The combination of pseudoephedrine and emodin can inhibit inflammatory pathways, alleviate pulmonary edema, decrease M1 macrophage polarization, and increase M2 macrophage polarization, significantly mitigating LPS-induced ALI in rats (34). Norephedrine possesses extensive applications in the treatment of various diseases. Clinically, oral norepinephrine exerts a modest decongestant effect in individuals with a cold; therefore, it is extensively utilized for the common cold (35). Phellamurin, a flavonoid glycoside found in plants, decreases the viability of osteosarcoma cells and promotes apoptosis by inhibiting the PI3K/AKT/mTOR pathway (36). 18β-GA is the main metabolite of glycyrrhetinic acid, which is the primary active component of licorice. 18β-GA exhibits a range of biological effects, including hepatic protective, anti-cancer, kidney protective, antiviral, antibacterial, and anti-inflammatory activities (37). Moreover, 18β-GA can downregulate the gene expression of *Icam-1*, *Tnf-Α*, *Cox-2*, and *iNos* in LPS-induced RAW 264.7 cells by decreasing NF-κB expression and inhibiting its nuclear translocation. It also downregulates the gene and protein expression levels of TNF-α, IL-6, and MCP-1 in the culture supernatant of human pulmonary artery smooth muscle cells stimulated by platelet-derived growth factor BB, demonstrating substantial anti-inflammatory efficacy (38, 39). Notably, DL-ephedrine, pseudoephedrine, and norepinephrine are all derived from ephedra, highlighting the pivotal role of ephedra in mediating the therapeutic effects of MXSGD against IAV infections.

The PPI network diagram illustrates several interactions among the identified targets. Our PPI network revealed 99 key targets. A higher degree of connectivity suggests a greater probability that MXSGD exerts its therapeutic effects against ALI through those specific targets. Among the 99 key targets, a substantial number were closely associated with macrophage polarization. For example, TNF-α, encoded by *Tnf*, is a classical pro-inflammatory factor that drives M1 macrophage polarization by activating multiple signaling pathways (40). IFN-γ, encoded by *Ifng*, promotes M1 polarization while suppressing M2 polarization via STAT pathway activation (41). COX-2, produced by *Ptgs2*, is implicated in inflammatory M1 polarization (42). The signaling pathways linked to these targets include the PI3K/AKT pathway, involving AKT1, PIK3R1, PIK3CA, and PTEN (43, 44); the MAPK pathway, involving MAPK1 and MAPK14 (45, 46); and the NF-κB pathway, mediated by IKBKB and CHUK (47). Notably, all these pathways are critical in the regulation of macrophage polarization. Furthermore, these key targets also encompassed transcription factors associated with macrophage polarization, including PPARG (48, 49), JUN, and FOS (50), as well as macrophage polarization-related targets such as NOS2. We then integrated the 99 key targets identified in the PPI network with the results from the CTP network and identified significant targets: EGFR, CDK2, MAPK1, GSTP1, MAPK8, TGFBR2, PIK3R2, AKT1, PIK3R1, and PIK3CA. Notably, all these targets exhibited strong binding affinities for blood components in the molecular docking analysis. GO and KEGG analyses indicated that the prospective targets of MXSGD in the treatment of ALI are predominantly enriched in the PI3K/AKT, MAPK, and nuclear receptor signaling pathways. For example, the PI3K/AKT signaling pathway is essential for cell survival, initiation of inflammatory responses, and oxidative stress in pulmonary diseases (51–53). This pathway affects the progression of various respiratory disorders, including ALI, ARDS, chronic obstructive pulmonary disease, asthma, and novel coronavirus pneumonia (51, 54–58). The primary targets linked to the regulation of the PI3K/AKT signaling pathway, *PIK3R1* and *PIK3R2*, encode the regulatory subunits p85α and p85β of PI3K, respectively, while *PIK3CA* encodes the catalytic subunit P110α of PI3K (59, 60). Moreover, the PI3K/AKT signaling pathway is closely associated with macrophage polarization. The activation of this pathway induces macrophage repolarization from the pro-inflammatory M1 phenotype to the anti-inflammatory M2 phenotype, thereby exerting anti-inflammatory effects (61). For example, grape seed proanthocyanidin alleviates LPS-induced ALI by inducing M1-to-M2a macrophage repolarization through the PI3K/AKT pathway (62). These results, along with network pharmacology and molecular docking findings, indirectly corroborate the accuracy of the network pharmacology-predicted targets.

Patients with ALI exhibit considerable accumulation of inflammatory cells in the lungs, which release inflammatory cytokines. These cytokines, in turn, activate various signaling pathways, forming a complex signaling network. Continuous stimulation by external antigens triggers an escalating pulmonary inflammatory response, which eventually becomes uncontrolled, leading to lung tissue damage (63–65). To further validate the network pharmacology and molecular docking results, we established an ALI mouse model induced by IAV and administered MXSGD to evaluate its effects on lung tissue pathology and inflammatory cytokines. Our findings demonstrated that MXSGD mitigated lung tissue damage, decreased viral load in the lungs, and downregulated the expression of IAV-induced inflammatory markers, thereby attenuating the pulmonary inflammatory response. Therefore, MXSGD exhibits favorable therapeutic efficacy.

Our network pharmacology analysis revealed that a significant proportion of key therapeutic targets of MXSGD against ALI are associated with macrophage polarization. Macrophages are key initiators of the inflammatory response, and alveolar macrophages play a central role in the pathogenesis of ALI, contributing to both inflammation and tissue repair (66). Alveolar macrophages function as antigen-presenting cells to trigger innate immunity and polarize into different phenotypes based on the local or systemic inflammatory microenvironment. After polarization, they can release large amounts of pro-inflammatory cytokines to drive the inflammatory response or anti-inflammatory cytokines to mediate the repair of damaged lung tissue. Therefore, we investigated macrophage polarization in mouse lung tissue treated with MXSGD. The gene expression of pro-inflammatory cytokines, including *Il-6* and *iNos*, was significantly downregulated following treatment with MXSGD. In contrast, the expression of the anti-inflammatory cytokines *Il-10* and *Fizz1* was markedly upregulated following MXSGD treatment. Therefore, we performed immunofluorescence double staining of macrophage polarization markers in mouse lung tissue to further elucidate the effects of MXSGD on macrophage polarization in ALI. Our results revealed that MXSGD significantly downregulated the expression of the pro-inflammatory M1 phenotype marker CD80 in IAV-infected lung tissue while simultaneously promoting the expression of the anti-inflammatory M2 phenotype marker CD206. These findings collectively suggest that MXSGD exerts protective effects against lung injury by modulating macrophage polarization, facilitating the transition from M1 to M2 phenotypes. Notably, this mechanistic insight is fully consistent with our network pharmacology predictions.

As mentioned previously, the PI3K/AKT signaling pathway plays a pivotal role in macrophage polarization, and substantial evidence highlights its regulatory functions. For example, aloe-emodin derivatives suppress macrophage inflammatory mediator release via PI3K-AKT/NF-κB signaling (67). Moreover, Xibining mitigate macrophage inflammation by regulating the PI3K/AKT pathway (68). Notably, our network pharmacology analysis revealed that the therapeutic targets of MXSGD for ALI were mainly enriched in PI3K/AKT pathway targets. Immunohistochemical analysis of lung tissues further confirmed that MXSGD intervention significantly downregulated IAV-induced PI3K/AKT pathway activation, validating the network pharmacology predictions. Nevertheless, whether MXSGD modulates macrophage polarization through PI3K/AKT suppression, as well as the precise mechanistic interplay between PI3K/AKT signaling and macrophage polarization in MXSGD-mediated ALI treatment, remains to be fully elucidated.

This study has certain limitations. First, as we did not quantify pharmacokinetic parameters, such as bioavailability and half-life, for the identified components, the main active components driving macrophage polarization and their direct molecular targets remain unclear. Second, although we preliminarily validated the involvement of the PI3K/AKT pathway, the specific upstream and downstream signaling molecules, tandem nodes, and their mechanism interactions need to be systematically defined. Therefore, future studies should screen the active, blood-absorbed components of MXSGD that target macrophage polarization to assess the necessity and sufficiency of PI3K/AKT signaling in cellular and animal models, as well as to elucidate the downstream regulatory mechanisms. Such studies will precisely identify the therapeutic mechanisms of MXSGD, provide strong scientific support for clinical optimization of the treatment of severe influenza-induced lung injury, and lay the foundation for rational drug combinations and TCM-based drug development.

In summary, we confirmed the significant efficacy of MXSGD in alleviating influenza-induced ALI and associated inflammation. Notably, through integrated network pharmacology and *in vivo* validation, we established macrophage polarization as a pivotal therapeutic mechanism. This discovery elucidates the scientific basis of MXSGD against influenza-associated ALI from an immunomodulatory perspective while providing mechanistic insights into the multi-component, multi-target, multi-pathway synergistic paradigm of TCM. We also identified the bioactive blood-absorbed components of MXSGD, offering a pharmacodynamic foundation for modern research and clinical applications.

## 5 Conclusion

In this study, we examined the blood-absorbed components of MXSGD, using network pharmacology and bioinformatics, to investigate the effective pharmacological components, key targets, and potential regulatory mechanisms underlying the therapeutic effects of MXSGD against ALI. Our findings provide new insights into the underlying mechanisms of MXSGD in treating ALI. However, further investigations are required to determine the mechanisms by which MXSGD regulates macrophage polarization to suppress inflammatory responses in the treatment of ALI.

## Data Availability

The original contributions presented in the study are included in the article/supplementary material. Further inquiries can be directed to the corresponding author.
